# Tumor Necrosis is Associated with an Increased Metastatic and Cardiovascular Risk in Paragangliomas: A Single-Center Series and Meta-Analysis Comparing Necrosis and Cystic Degeneration in Paragangliomas

**DOI:** 10.1007/s12022-026-09931-1

**Published:** 2026-08-22

**Authors:** Anna-Maria Wiejak, Georgiana Constantinescu, Gintare Zygiene, Mercedes Robledo, Christian G. Ziegler, Jens-Peter Kühn, Ronald de Krijger, Christina Pamporaki, Matthias Meinhardt, Nicole Bechmann

**Affiliations:** 1 Department of Medicine III, University Hospital Carl Gustav Carus, Technical University Dresden, Dresden, Germany; 2 Grigore T Popa University of Medicine and Pharmacy, Iasi, Romania; 3 Institute for Clinical Chemistry and Laboratory Medicine, Medical Faculty Carl Gustav Carus, University Hospital Carl Gustav Carus, Technische Universität Dresden, Dresden, Germany; 4 Hereditary Endocrine Cancer Group, Human Cancer Genetics Program, Spanish National Cancer Research Centre (CNIO), Madrid, Spain; 5 Centro de Investigación Biomédica en Red de Enfermedades Raras (CIBERER), Institute of Health Carlos III (ISCIII), Madrid, Spain; 6 Institute and Policlinic for Diagnostic and Interventional Radiology, Faculty of Medicine, University Hospital Carl Gustav Carus, Technische Universität Dresden, Dresden, Germany; 7 Princess Máxima Center for Pediatric Oncology, Utrecht, the Netherlands; 8 Department of Pathology, University Medical Center Utrecht, Utrecht, the Netherlands; 9 Department of Pathology, Faculty of Medicine Carl Gustav Carus, Faculty of Medicine Carl Gustav Carus, Technical University Dresden, Technical University Dresden, Dresden, Dresden, Germany; 10 Experimental Endocrinology and tumor cell metabolism, Medical School OWL, Bielefeld University, Bielefeld, Germany

**Keywords:** Metastasis, Hemorrhagic alterations, Neuroendocrine tumors, Necrosis, Cardiovascular complications, Preoperative management

## Abstract

Paragangliomas (PGLs) are rare neural crest–derived tumors with highly variable morphology and clinical behavior. These tumors can present with cystic and necrotic alterations that may influence the clinical presentation of affected patients. However, prevalence and clinical significance of these pathological alterations remain poorly understood. In this retrospective study (2011–2021), 103 patients with confirmed PGLs were examined for cystic and necrotic alterations based on imaging and pathology reports as well as pathological re-evaluation. Cystic alterations, including microscopic, macroscopic, and pseudocystic alterations, were observed in 61.8% of PGLs. Regardless of their pathomorphological presentation, cystic alterations did not affect the clinical presentation of these patients. In contrast, necrotic alterations were identified in 3.9% of PGLs and were associated with a higher rate of metastasis (75% vs. 10%, *p* = 0.0124), larger tumor volume (53.6 cm^3^ vs. 6.6 cm^3^*p* = 0.0339), and more pronounced signs and symptoms, including hypertension, palpitations, and weakness compared to patients without cystic/necrotic lesions. The prevalence of cystic and necrotic lesions was higher in adrenal than extra-adrenal PGLs. An additional meta-analysis of 235 patients with PGLs confirmed the increased rate of metastasis in necrotic PGLs (16% vs. 4.5%, *p* = 0.0369), which were also significantly more frequently associated with cardiovascular complications (*p* < 0.0001) than cystic PGLs. Considering pathological alterations, particularly necrotic lesions, may substantially affect the clinical management of patients with PGLs. Incorporating these features into preoperative management and follow-up strategies might be crucial, given the higher metastatic potential and increased cardiovascular risk associated with necrotic PGLs.

**Supplementary Information:**

The online version contains supplementary material available at 10.1007/s12022-026-09931-1.

## Introduction

Paragangliomas (PGLs) are rare neoplasms that arise from neural-crest derived cells of the sympathetic or parasympathetic ganglia and are characterized by a distinct clinical presentation [1]. PGLs of the adrenal medulla are also referred to as pheochromocytomas. PGLs are often discovered incidentally during imaging examinations performed for causes unrelated to adrenal masses, or because patients present with signs and symptoms related to the catecholamine excess of these tumors. The main clinical signs and symptoms of PGLs include recurrent paroxysms of hypertension, headache, palpitations, diaphoresis and pallor, but anxiety, tremor, nausea, vomiting, and weight loss should also be considered [2, 3]. Excessive, continuous, or paroxysmal catecholamine secretion by these tumors can lead to life-threatening cardiovascular complications.

Based on their transcriptional profile, PGLs are divided into three main clusters associated with specific phenotypic features [4, 5]. Cluster 1 PGLs are characterized by continuous activation of hypoxia signaling pathways due to pathogenic variants (PVs) in e.g. succinate dehydrogenase subunits A-D (*SDHx*), fumarate hydratase (*FH*), and von Hippel-Lindau (*VHL*) tumor suppressor. Cluster 1 PGLs are at higher risk to metastasize and are distinguished by a more immature catecholamine phenotype compared with cluster 2 PGLs [6, 7]. Cluster 2 PGLs exhibit PVs in genes that lead to activation of kinase signaling pathways, such as RET protooncogene (*RET*) and neurofibromin 1 (*NF1*) [5]. In Caucasians, cluster 2 PGLs occur almost exclusively in the adrenal, whereas cluster 1 PGLs can occur at adrenal- and extra-adrenal locations [8]. The catecholamine phenotype is determined by the underlying PV and the access to adrenal cortical glucocorticoids, which induce the expression of phenylethanolamine *N*-methyltransferase (PNMT) [8, 9]. Cluster 2 PGLs are capable to produce adrenaline through the PNMT-mediated conversion of noradrenaline to adrenaline, whereas cluster 1 PGLs lack PNMT expression and are predominantly noradrenergic [10]. Cluster 3 are characterized by activation of Wnt/β-catenin signaling, typically driven by *MAML3* rearrangements or *CSDE1* mutations, and are associated with a more aggressive clinical phenotype [11].

All PGLs carry a risk of metastasis [1], but effective predictive markers are still lacking. Several molecular, genetic and biochemical markers are discussed, but usually only a combination of different markers provides a predictive assessment for metastatic disease [12]. The histopathological scores (e.g., PASS [13] and GAPP [14] score) discussed to assess metastatic potential of PGLs take the presence of necrosis into account. However, necrosis alone is not sufficient to reliably predict the metastatic potential of PGL, but in combination with other features, it is considered a worrisome histopathological feature. The prevalence of necrosis in PGLs and whether it correlates with certain clinical and phenotypic features remains unclear. PGL tumor cell spheroids, which recapitulate micro-metastases, suggests that necrotic changes in the inner core of these 3D-structures lead to accumulation of catecholamines [15]. Moreover, the appearance and frequency of cystic alterations in PGL vary based on imaging studies, while focal cystic alterations (focal regions of low attenuation that appear to have fluid density) appear relatively often (55% [16]), only around 3% of patients present with heterogeneous cystic lesions with a thick solid rim [17]. New insights into clinical consequences of such regressive and haemorrhagic alterations in PGLs might be crucial for making valid decisions, for example, whether a patient would benefit from preoperative blockade with α-adrenoreceptor antagonists [18].

Primary objective of this study was to determine the prevalence of cystic and necrotic alterations in PGLs and to investigate possible clinical implications in a single-center cohort. We investigated whether various parameters, such as patient sex, tumor volume, or specific biochemical phenotypes, are associated with an increased occurrence of cystic or/and necrotic lesions and examined whether these pathological alterations correlated with distinct clinical features.

## Materials and Methods

### Subjects

**Fig. 1:**
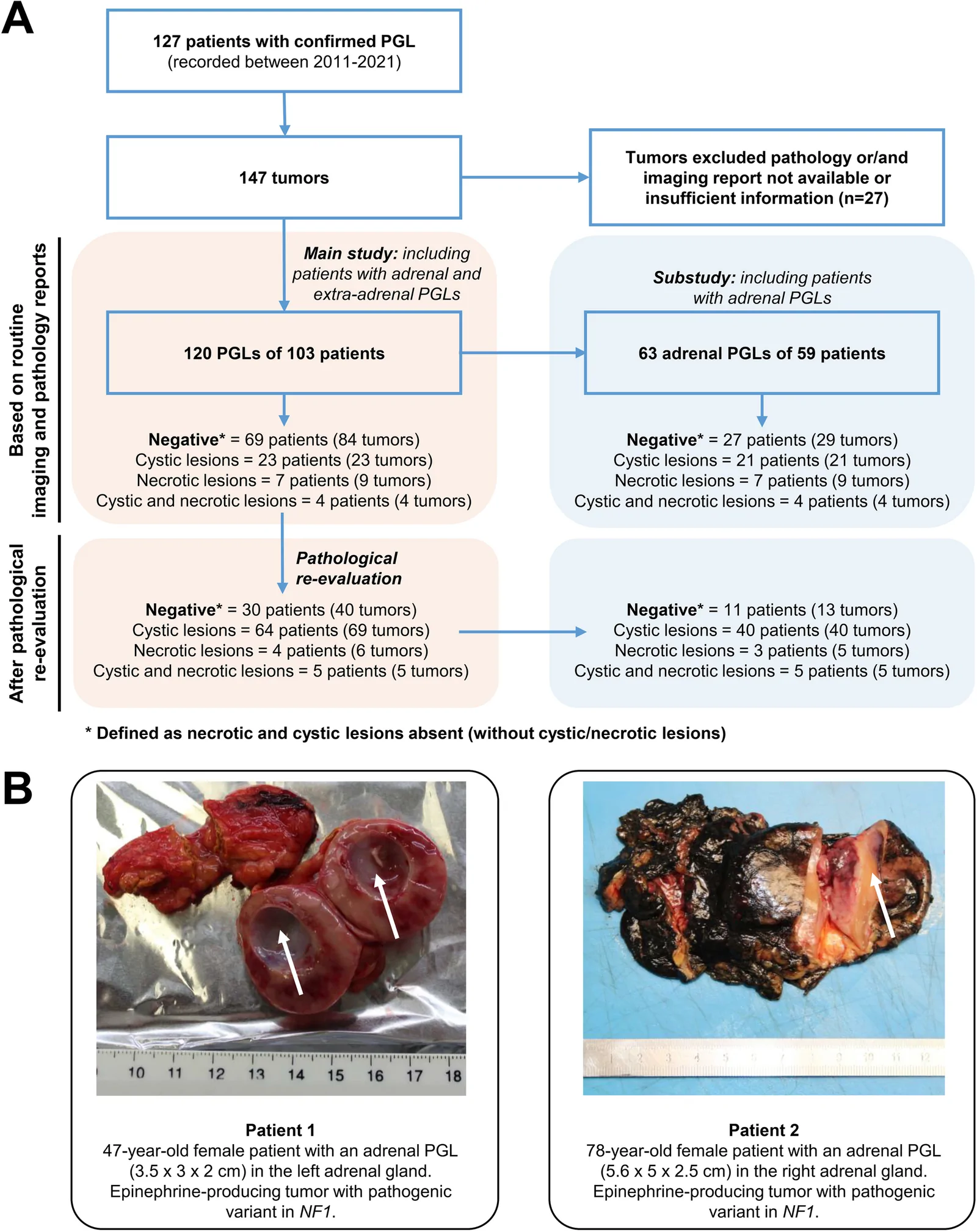
Flow diagram regarding study inclusion and examples of adrenal paragangliomas (PGLs) with macroscopic cysts. (**A**) Study work flow including exclusion criteria. (**B**) Two examples of adrenal PGLs presenting with macroscopic cysts

We retrieved data from 127 patients diagnosed with PGL. Patients recruited at the University Hospital Carl Gustav Carus Dresden between January 2011–September 2021 and enrolled under the European Network for the Study of Adrenal Tumours (ENSAT; EK407122010) protocol and/or Prospective Monoamine-Producing Tumor study (PMT; EK189062010) or Prospective Pheochromocytoma Study (PROSPHEO; EK210052017) were included in the present study. All patients gave written informed consent before enrollment under respective study protocols. Local ethics committees approved all study protocols. Of the 127 patients (147 tumors), we excluded 24 patients (27 tumors) due to missing imaging/pathology reports, or insufficient data (Fig. 1). Information regarding clinical data assessment, biochemical analysis and genetic testing were given in the supplemental material 2. Metastatic disease was defined as the presence of metastases in tissues distant from the primary tumor where chromaffin cells are normally absent. Metastatic disease was confirmed by conventional and functional imaging or occasionally by histopathological examination of resected lymph nodes.

### Assessment of Cystic and Necrotic Lesions by Routine Imaging and Pathology Reports

Routine imaging and pathology reports were reviewed for cystic and/or necrotic lesions, which were recorded only when explicitly stated. Imaging assessment was based on magnetic resonance imaging (MRI) and computed tomography (CT) reports by board-certified radiologists. Other imaging modalities were not included. On MRI, both appear hyperintense on T2-weighted images with differentiation supported by contrast enhancement, while on CT both typically present as hypodense regions. Only explicitly stated cystic or necrotic lesions in imaging/pathology reports were considered. Descriptions suggesting cystic or necrotic changes (e.g., “regressive”, “pseudocystic”, “hypodense area” or “semi-liquid parts”) were excluded and classified as “negative” or “without cystic/necrotic lesion”.

### Pathological Re-Evaluation

For 87 PGLs, hematoxylin&eosin-stained sections were available from the Department of Pathology (University Hospital Carl Gustav Carus). Pathological re-evaluation was performed by two independent examiners (M.M. and A.M.W.), which rated histological sections by defined criteria. For each PGL a maximum of six tumor sections were evaluated. For patients where no histological sections were available, assessment was based on pathology or radiology findings. Pathological re-evaluation was primarily intended to incorporate smaller pseudocysts, which might not be not recorded in routine reports.

### Evaluation of Macroscopic Cysts

Overview images of tumors immediately after resection were available for 45 patients (46 tumors). These were assessed for the presence of macroscopic cysts by N.B. (Fig. 1).

### Meta-Analysis

**Fig. 2:**
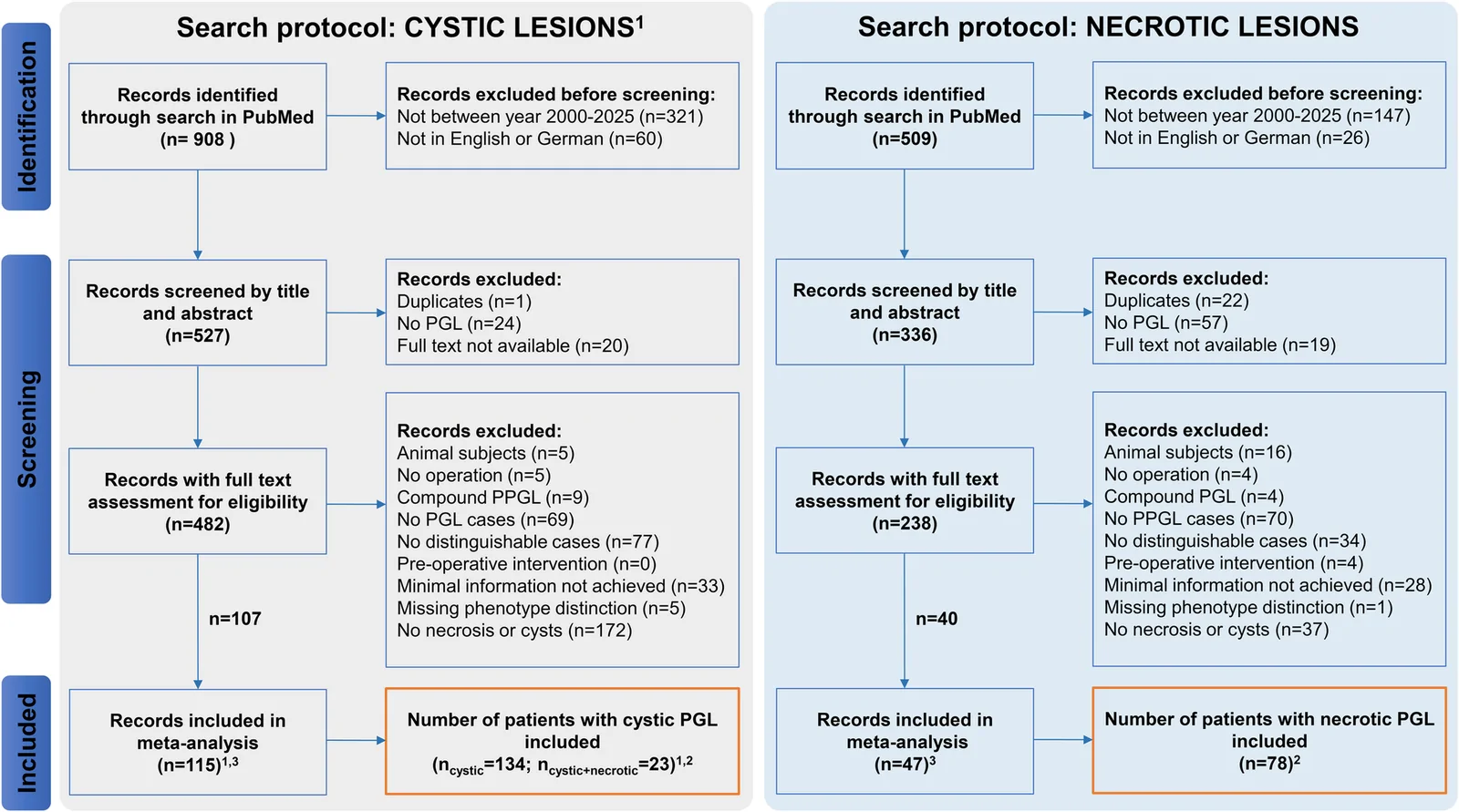
PRISMA flow diagram about study inclusion for meta-analysis of cystic and necrotic paragangliomas (PGLs). ^1^Including patients described with cystic lesions alone and with cystic and necrotic lesions; ^2^Patients with bilateral disease or multiple tumors included; ^3^Additional records/publications were identified (n_necrotic_=7; n_cystic_=8), in addition to the performed PUBMED search

We conducted a search in PUBMED with two search queries for cystic and necrotic PGLs respectively (last search: 13.06.2025). Details were provided in supplemental material 1. Case reports and publications on human subjects were eligible if they reported at least minimal clinical and pathological information, including age, sex, tumor location, and the presence of cystic or/and necrotic lesions, and were available in English or German. The search term yielded in 908 (SEARCH 1) and 509 (SEARCH 2) publications that were evaluated for suitability by A.M.W. and N.B. following defined criteria (Fig. 2 and supplementary material 2).

### Statistics

The statistical analyses were carried out using JMP Student Edition 18 (SAS Institute) and GraphPad Prism 10 (Siemens Digital Industries Software). Continuous data were tested for normal distribution using the Shapiro-Wilk test. Mean values of continuous data were compared with the Kruskal-Wallis rank sum test or unpaired t-test. A Pearson chi-square test with contingency table and Fisher´s exact test was performed to compare the frequency of necrotic lesions in males and females, and their association with metastatic disease and certain signs and symptoms, with PGLs without cystic/necrotic lesions.

## Results

### Clinical Characteristics of Patients with Cystic PGL

In our single-center cohort of 103 patients (59.2% females) with confirmed PGLs (n_tumor_=120), cystic or/and necrotic lesions were described in pathology or/and imaging reports of 34 patients (33.0%, Table 1). Cystic lesions were reported in 23 cases (22.3%). The median age at operation of patients with cystic lesions was 46.5 years (range: 9–74) and 13 patients (56.5%) were female. Of the 23 PGLs with cystic lesions, 21 (91.3%) presented within the adrenal and two as extra-adrenal PGLs (including one head and neck PGL (HNPGL)), with none presenting with metastatic disease (Table 1, and S1-2). No significant difference in mode of discovery (incidentally vs. catecholamine-related signs and symptoms vs. other conditions) could be found between cystic PGLs and PGLs without cystic/necrotic lesions.

**Table 1:** Characteristics of patients with paraganglioma in dependence of occurrence of cystic or/and necrotic lesions reported by routine radiological and/or pathological evaluation

	Total	Negative ^a^	Cystic lesions	Necrotic lesions	Cystic and necrotic lesions
Number of patients *n* ([%])	103 (100)	69 (67)	23 (22.3)	7 (6.8)	4 (3.9)
Number of tumors n ([%])	120 (100)	84 (70)	23 (19.2)	9 (7.5)	4 (3.3)
**Sex**^**1**^					
Female n ([%]) Male n ([%])	61 (59.2) 42 (40.8)	46 (66.7) 23 (33.3)	13 (56.5) 10 (43.5) *p* > 0.05	1 (14.3) 6 (85.7) *p* = 0.0109^b^	1 (25) 3 (75)
**Age at operation**^**1**^					
Mean ± SD [years] Median (range) [years]	48.1 ± 17.0 50 (9–79)	48.3 ± 16.8 51 (11–79)	46.5 ± 18.1 46.5 (9–74) *p* > 0.05^e^	44.4 ± 17.7 51 (12–64) *p* > 0.05^e^	60.8 ± 12.3 59.5 (47–77)
**Tumor volume** Mean ± SD [cm^3^] Median (range) [cm^3^]	118 51.1 ± 113.0 15.9 (0.004–904.9)	82 23.9 ± 42.4 8.5 (0.004–245.1)	23 99.2 ± 187.9 42.4 (0.7–904.9) *p* > 0.05^e^	9 151.1 ± 204.9 82.5 (18.9–659.8) *p* = 0.0009^**e**^	4 107.1 ± 77.6 99.0 (20.9–209.5)
**Localization**^**2**^					
A: EA: HN: A + EA	62:16:41:1	28:15:40:1	21:1:1:0	9:0:0:0	4:0:0:0
**Cysts/Necrosis described in:**					
imaging report n ([%]) pathology report n ([%]) both n ([%]) not mentioned n ([%])	6 (5) 18 (15) 12 (10) 84 (70)	0 0 0 84 (100)	2 (8.7) 10 (43.5) 11 (47.8) 0	3 (33.3) 5 (55.6) 1 (11.1) 0	1 (25) 3 (75) 0 0
**Metastatic disease**^**1**^ yes: no n ([%])	8(7.8):95(92.2)	5(7.2):64(92.8)	0(0):23(100) *p* > 0.05^b^	3(42.9):4(57,1) *p* = 0.0225^**b**^	0(0):4(100)
**Mode of discovery n ([%)]**					
**incidentally** **catecholamine-related SS** **post-OP follow-up** **genetic surveillance** **other reasons or no information**	64 (62.1) 30 (29.1) 1 (1.0) 4 (3.9) 4 (3.9)	47 (68.2) 16 (23.2) 1 (1.4) 4 (5.8) 1 (1.4)	13 (56.5)^b^ 8 (34.8)^b^ 0 0 2 (8.7)	4 (57.1)^b^ 3 (42.9)^b^ 0 0 0	0 3 (75) 0 0 1 (25)
**Pathogenic variants**^**1,3**^ n ([%])	*CDKN1B*: 1 (1.1) *EPAS1*: 2 (2.3) *FGFR1*: 1 (1.1) *FH*: 1 (1.1) *HRAS*: 5 (5.6) *KIF1B*:1 (1.1) *NF1*: 13 (14.8) *RET*: 5 (5.7) *SDHA*: 2 (2.3) *SDHAF2*: 1 (1.1) *SDHB*: 6 (6.8) *SDHC*^d^: 3 (3.4) *SDHD*: 6 (6.8) *VHL*: 6 (6.8) negative: 35 no information available: 15 Cluster 1: 27 (52.9) Cluster 2: 24 (47.1)	*CDKN1B*: 1 (1.8) *EPAS1*: 1 (1.8) *FH*: 1 (1.8) *HRAS*: 3 (5.3) *NF1*: 8 (14.0) *RET*: 1 (1.8) *SDHA*: 2 (3.5) *SDHAF2*: 1 (1.8) *SDHB*: 6 (10.5) *SDHC*^d^: 2 (3.5) *SDHD*: 6 (10.5) *VHL*: 3 (5.3) negative: 22 no information available: 12 Cluster 1: 22 (64.7) Cluster 2: 12 (35.3)	*EPAS1*: 1 (4.5) *FGFR1*: 1 (4.5) *HRAS*: 1 (4.5) *KIF1B*:1 (4.5) *NF1*: 3 (13.6) *RET*: 4 (18.2) *VHL*: 2 (9.1) negative: 9 no information available: 1 Cluster 1: 3 (25) Cluster 2: 9 (75)	*NF1*: 1 (20) *SDHC*: 1 (20) negative: 3 no information available: 2 Cluster 1: 1 (50) Cluster 2: 1 (50)	*HRAS*: 1 (25) *NF1*: 1 (25) *VHL*: 1 (25) negative: 1 no information available: - Cluster 1: 1 (33.3) Cluster 2: 2 (66.7)
**Signs and symptoms**^**1**^					
Hypertension n ([%]) Headaches n ([%]) Sweatiness n ([%]) Palpitations n ([%]) Tremor n ([%]) Pallor n ([%]) Flushing n ([%]) Panic n ([%]) Nausea n ([%]) Weakness n ([%]) Abdomen pain n ([%]) Chest pain n ([%]) Constipation n ([%]) Paroxysmal HP n ([%]) Diabetes n ([%])	52/69 (75.4) 34/66 (51.5) 33/64 (51.6) 22/64 (34.4) 7/64 (10.9) 13/64 (20.3) 14/64 (21.9) 15/64 (23.4) 11/64 (17.2) 20/64 (31.2) 8/64 (12.5) 7/65 (10.8) 5/64 (7.8) 23/65 (35.4) 16/71 (22.5)	29/39 (74.4) 20/38 (52.6) 17/36 (47.2) 10/36 (27.7) 4/36 (11.1) 9/36 (25) 8/36 (22.2) 8/36 (22.2) 7/36 (19.4) 11/36 (30.6) 4/36 (11.1) 4/36 (11.1) 4/36 (11.1) 12/36 (33.3) 9/41 (22.0)	14/20 (70.0) 10/19 (52.6) 11/19 (57.9) 8/19 (42.1) 3/19 (15.8) 3/19 (15.8) 4/19 (21.1) 6/19 (31.6) 3/19 (15.8) 4/19 (21.1) 3/19 (15.8) 3/19 (15.8) 1/19 (5.2) 6/19 (31.6) 4/20 (20.0)	6/6 (100) 3/6 (50) **4/6 (66.6)** **4/6 (66.6)** 0/6 (0) 1/6 (16.7) 2/6 (33.3) 1/6 (16.7) 1/6 (16.7) **4/6 (66.7)** 1/6 (16.7) 0/6 (0) 0/6 (0) **3/6 (50)** 2/6 (33.3)	3/4 (75) 1/3 (33.3) 1/3 (33.3) 0/3 (0) 0/3 (0) 0/3 (0) 0/3 (0) 0/3 (0) 0/3 (0) 1/3 (33.3) 0/3 (0) 0/3 (0) 0/3 (0) 2/4 (50) 1/4 (25)

^1^ Patients with multiple tumors where information regarding all tumors were available (*n* = 4) are only included once in the analysis

^2^ Including bilateral and multiple tumors

^3^ Patients tested for pathogenic variants (germline and/or somatic testing)

^a^ No cystic or necrotic alterations described by imaging and pathological report

^b^ ChiSquare test with Fisher´s Exact test compared to negative PGLs

^c^ Two of the three metastatic cases were male

^d^ Including one case with *SDHC* promoter methylation

^e^ Kruskal-Wallis rank sum test lesions compared to negative PGLs

A: adrenal; EA: extra-adrenal; HN: head & neck; HP: hypertension; SS: signs and symptoms

Patients with cystic PGL were operated at similar age compared to patients without cystic/necrotic lesions (46.5 vs. 51 years). In both groups, the proportion of females predominated (56.5% and 66.7% females, female-to-male ratio: 1.3:1 and 2:1), although not significant. PGLs with cystic lesions presented in trend with a larger tumor volume compared to those without cystic/necrotic lesions (42.4 vs. 8.5 cm^3^). None of the patients with cystic PGLs had metastatic disease, while 7.2% of the cohort without cystic/necrotic lesions presented with metastatic disease (Table 1, and S3). Cystic lesions occurred in PGLs with PVs in *VHL*, *EPAS1*, *NF1*, *RET*, *HRAS*, *FGFR1*, and *KIF1B* (25% cluster 1 vs. 75% cluster 2 PGLs).

**Fig. 3:**
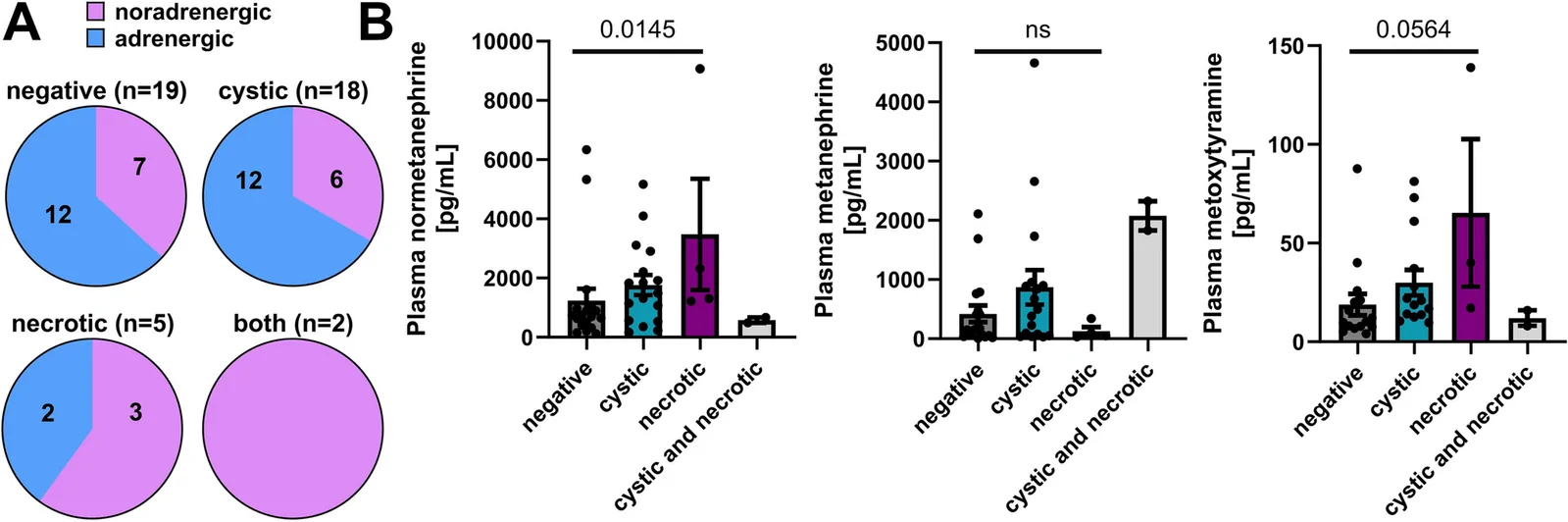
Biochemical phenotype of cystic or necrotic adrenal paragangliomas (PGLs). (**A**) Catecholamine phenotype (noradrenergic versus adrenergic phenotype) in patients with or without cystic or necrotic tumor lesions. (**B**) Plasma metanephrine levels of patients with cystic or/and necrotic adrenal PGLs

The biochemical phenotype of PGLs is strongly related to tumor location and extra-adrenal PGLs almost exclusively present with a dopaminergic/noradrenergic phenotype [7]. Since most of the cases in the present study occurred at adrenal location, we next investigated the catecholamine phenotype of patients with adrenal PGLs. Patients with cystic lesions more often presented with an adrenergic than a noradrenergic phenotype (Fig. 3A). No difference in plasma metanephrine levels between patients with adrenal PGLs with cystic lesions and those without cystic/necrotic lesions were observed (Fig. 3B).

The occurrence of cystic lesions was not associated with an increased number of clinical signs and symptoms in comparison to PGLs without cystic/necrotic lesions, regardless of tumor location (Table 1, and S1-2). The presence of cystic lesions appears to have no influence on the clinical and biochemical presentation of PGLs.

### Clinical Characteristics of Patients with Necrotic PGL

Necrotic lesions were reported in seven patients with PGL (two bilateral cases; n_tumor_= 9), representing 6.8% of all patients with PGL in our cohort (Table 1). The occurrence of necrotic lesions was only described in PGLs with adrenal location (Table S1−2). Patients with necrotic lesions had a median age of 51 years at the time of surgery (range: 12–64) and thus were operated at a similar age as patients without cystic/necrotic lesions (51 years; range: 11–79; Table 1). In contrast to patients without cystic/necrotic lesions, male sex predominated in patients with necrotic PGLs (female-to-male ratio: 0.17:1; *p* = 0.0109). PGLs with necrotic lesions were significantly larger than PGLs without cystic/necrotic lesions (82.5 vs. 8.5 cm^3^; *p* = 0.0009). Patients with necrotic PGLs presented significantly more often with metastatic disease (Table 1, and S3) than patients without cystic/necrotic lesions (42.9% vs. 7.2%, *p* = 0.0225). Of the three patients with metastatic disease, two were males. Only in two patients with necrotic lesions PVs were detected in known PGL susceptibility genes (1 *SDHC* promotor methylation, 1 *NF1*).

Patients with necrotic PGLs were in trend more often diagnosed due to catecholamine-related signs and symptoms than patients without cystic/necrotic lesions (42.9% vs. 23.2%, not significant). Three of five patients presented with a noradrenergic phenotype (Fig. 3A). Compared to patients with PGL without cystic/necrotic lesions, those with necrotic lesions had higher levels of normetanephrine (*p* = 0.0145) and there was a trend towards elevated methoxytyramine (*p* = 0.0564), while metanephrine levels tended to be lower (Fig. 3B). Patients with necrotic PGL present more often with sweating (66.7%), palpitations (66.7%), weakness (66.7%) compared to patients without cystic/necrotic lesions (47.2, 27.7, and 30.6%, Table 1).

Moreover, four PGL cases with cystic and necrotic lesions (3.9%, female-to-male ratio: 0.3:1) have been identified, who showed no notable differences in the clinical presentation compared to PGLs without cystic/necrotic lesions (Table 1).

### Pathological Re-Evaluation of Cystic and Necrotic Lesions in PGLs

**Fig. 4:**
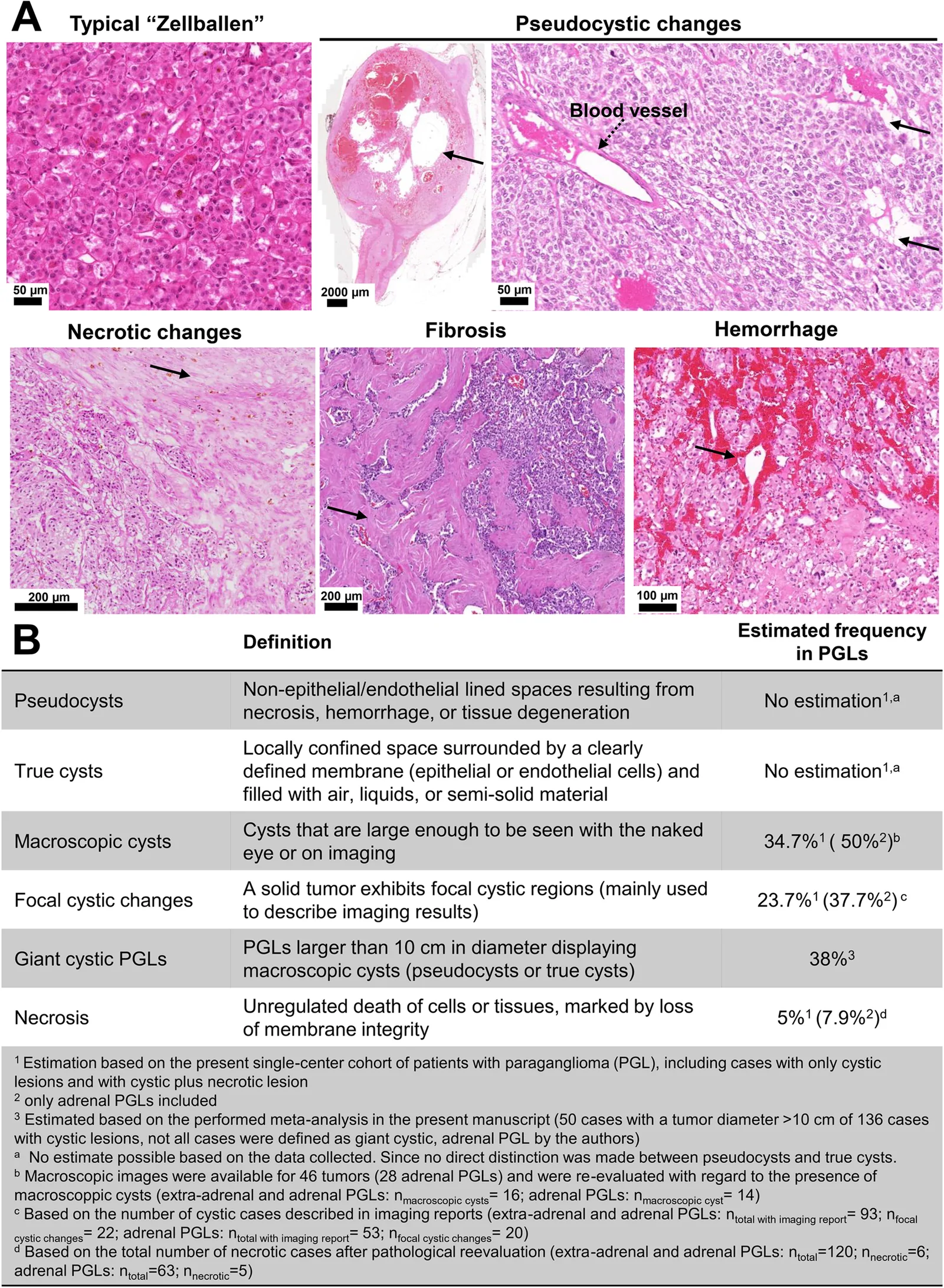
Histological features and proposed terminology, as well as observed frequencies for cystic changes in paragangliomas (PGLs). (**A**) Examples of histological features in PGLs, including the characteristic “Zellballen”, pseudocystic and necrotic changes, fibrosis, and hemorrhages. Continuous arrows indicate the histological features of the respective image. (**B**) Proposed definitions and observed frequencies for terms used in the literature to describe cystic changes in PGLs. The frequencies indicated refer to the data collected in the present paper

As cystic and necrotic lesions were not systematically documented in imaging and pathology reports, and given the inconsistencies in the terminology used, a pathological re-evaluation of available tissue sections was performed. This also allowed for precise identification of PGLs with small pseudocystic changes. In addition to the typical “Zellballen” of PGLs, fibrosis and hemorrhage were observed (Fig. 4A); however, these were not evaluated further. The number of PGLs classified as cystic (n_cystic tumors_=69) increased with the pathological re-evaluation, rising from 19.2% to 57.5% (Table 1 and S4-7). Prevalence of PGLs with necrotic lesions decreased from 7.5% to 5%. Some of the findings from imaging and pathology reports could not be confirmed, either due to misclassification or inaccurate information documented. Clinical characteristics between cystic PGLs and PGLs without cystic/necrotic lesions remained comparable even after re-evaluation (Table S4-7). Despite the reduced prevalence of necrotic lesions, an additional case of a necrotic *SDHB*-mutated HNPGL was identified (Table S4-7). Compared to PGLs without cystic/necrotic lesions, those with necrosis presented overall with larger tumor volume (53.6 vs. 6.6 cm^3^, *p* = 0.0339) and increased metastasis rate (75% vs. 10%, *p* = 0.0124, Table S3−4). All three adrenal PGLs, where the necrosis was pathologically confirmed, presented with metastases (*p* = 0.0110); in two of these cases no PV was detected, and in the third case, no genetic testing was performed (Table S4-7).

### Meta-Analysis of Patients with Cystic and/or Necrotic PGLs

To further characterize patients with cystic and/or necrotic lesions, we next performed a meta-analysis. In total, 235 patients diagnosed with PGL (n_tumors_=239) were identified with cystic (n_tumors_=136; [16, 17, 19–109]), necrotic (n_tumors_=79; [35, 110–155]), or cystic and necrotic (n_tumors_=24; [104, 156–177]) lesions in the literature from 2000 to June 2025 (Table 2, and S8-9). In the total cohort, patients were predominantly female (60.4%) with a median age of 49 years (3–86 years). In contrast to our single center cohort, extra-adrenal PGLs (n_PGL_=21; 26.6%), including HNPGLs (n_HNPGL_=7; 8.9%), with necrotic lesions have also been described in the literature. Patients with necrotic PGLs were operated at younger age than patients with cystic PGLs (44 vs. 52 years; *p* < 0.0001). Unlike in our cohort, tumor volume in PGLs with cystic lesions was significantly larger than in necrotic cases (288.7 vs. 65.5 cm^3^; *p* < 0.0001). PGLs with necrotic lesions had a higher rate of metastasis than those with cystic lesions (16.0% vs. 4.5%; *p* = 0.0369). The rates of cases with intraoperative complications were comparable between both groups. However, PGLs with necrotic lesions were more likely to present with cardiovascular complications than cystic PGLs (58.1% vs. 16.7%; *p* < 0.0001; Table 2). PGLs with necrotic lesions were discovered more often due to catecholamine-related signs and symptoms than PGLs with cystic lesions (59.0% vs. 29.0%; *p* = 0.0016). Patients with cystic and necrotic PGLs exhibit characteristics similar to those with cystic lesions only. Similar results were obtained when analysis was restricted to adrenal PGLs (Table S8 and S11).

**Table 2:** Characteristics of patients with paragangliomas in dependence of the occurrence of cystic or/and necrotic lesions reported in literature – Meta-analysis

	total	Cystic lesions	Necrotic lesions	Cystic and necrotic lesions
Number of tumors *n* ([%])	239 (100)	136 (56.9)	79 (33.1)	24 (10)
Number of patients n ([%])	235 (100)	134 (57.0)	78 (33.2)	23 (9.8)
**Sex**^**1**^				
Female n ([%]) Male n ([%])	142 (60.4) 93 (39.6)	81 (60.4) 53 (39.6)	47 (60.3) 31 (39.7)	14 (60.9) 9 (39.1)
**Age**^**1**^ Mean ± SD [years] Median (range) [years]	47.0 ± 16.4 49 (3–86)	50.8 ± 15.8 52 (4–86)	41.4 ± 16.0 44 (9–80) *p* < 0.0001^b^	43.2 ± 15.8 46 (3–64) *p* = 0.0440^b^
**Localization**^**1**^				
A: EA: HN	174:52:9	104:28:2	50:21:7	20:3:0
**Tumor side (adrenal PGLs)**				
left: right: bilateral: unknown^1^	66:76:7:25	33:42:5:24	23:26:1:1	10:9:1:0
**Tumor volume [n]** Total, mean ± SD [cm^3^] Total, median (range) [cm^3^]	177 602.8 ± 1110.5 189.4 (4.8–10307.2)	115 744.5 ± 1286.4 288.7 (4.8 −10307.2)	38 114.8 ± 131.7 65.5 (9.2–477.9 *p* < 0.0001^b^	24 696.3 ± 839.5 379.4 (31.7–3002.1) *p* > 0.05^b^
**Metastatic disease**^**1**^ yes: no: unknown n ([%])^1^	13(9.8):120(90.2):102	3(4.5):64(95.5):67	8(16):42(84):28 *p* = 0.0369^a^	2(12.5):14(87.5):7 *p* > 0.05^a^
**Mode of discovery** n ([%])				
incidentally Catecholamine-related SS insufficient information	103 (64.4) 57 (35.6) 79	71 (71) 29 (29) 36	16 (41.0; *p* = 0.0016^a^) 23 (59.0; *p* = 0.0016^a^) 40	16 (76.2) 5 (23.8) 3
**Pathogenic variants**^**1,2**^				
*SDHB* *VHL* *RET* *NF1*	1 5 6 4	0 3 5 1	1 1 0 1	0 1 1 2
**Cardiovascular complications**^**1**^ yes: no: unknown n ([%])	34(31.2):75(68.8):126	10(16.4):51(83.6):73	18(58.1):13(41.9):47 *p* < 0.0001^a^	6(35.3):11(64.7):6 *p* > 0.05^a^
**Intraoperative complications**^**1**^ yes: no: unknown n ([%])	29(46.0):34(54.0):172	21(45.7):25(54:3):88	1(16.7):5(83.3):73 *p* > 0.05^a^	7(63.6):4(36.4):12 *p* > 0.05^a^
**Patient alive**^**1,3**^ yes: no: unknown n ([%])	166(93.8):11(6.2):58	82(95.3):4(4.7):48	63(91.3):6(8.7):9 *p* > 0.05^a^	21(95.4):1(4.6):1 *p* > 0.05^a^

^1^ Bilateral case where information regarding both tumors were available (*n* = 4) are only included once in the analysis

^2^ For most of the cases no information about pathogenic variants were available

^3^ Within the follow-up period of the respective paper

^a^ ChiSquare test with Fisher´s Exact test compared to cystic lesions

^b^ Kruskal-Wallis rank sum test compared to cystic lesions

A: adrenal; EA: extra-adrenal; HN: head & neck; SS: signs and symptoms

### Terminology and Frequencies of Cystic Lesions in PGLs

The evaluation of the literature and screening of pathology and radiology reports revealed that the term “cysts” was applied inconsistently, and it was often unclear what type of intra-tumoral change had been observed. Based on our expertise, we proposed the following definitions in the context of PGLs and calculated the respective frequencies based on our data (Fig. 4). “Pseudocysts” were defined as non-epithelial/endothelial lined spaces resulting from necrosis, hemorrhage, or tissue degeneration. Based on our data, no distinction between pseudocysts and true cysts, defined as localized spaces surrounded by a clearly defined membrane and filled with air, fluids, or semi-solid substances, was possible. “Macroscopic cysts” were defined as cysts that are large enough to be seen with the naked eye or on imaging. In the present cohort, macroscopic cysts were present in 34.7% of PGLs (adrenal PGLs: 50%; Figs. 1 and 4). Additionally, the term “focal cystic changes” was used in literature to describe a solid tumor that exhibits focal cystic regions, which was mainly used to describe imaging results. In our cohort 23.7% of PGLs (37.7% of adrenal PGLs) presented with focal cystic changes estimated by imaging reports. For “giant cystic pheochromocytoma” no clear cut-off was defined, but in the performed meta-analysis, most giant cystic PGLs [36, 37, 42, 45, 48, 49, 51, 63, 70, 71, 73, 77, 78, 83, 84, 87–89, 95, 100, 106, 107, 156, 163, 167, 171, 173] described were > 10 cm in diameter (15.5 cm; range: 9.8–27 cm) and displayed macroscopic cysts. In the cystic PGL cohort, 38% met criteria of giant cystic PGLs. Necrosis referred to the unregulated death of cells or tissue, which is marked by a loss of membrane integrity (Fig. 4). The frequency of necrotic lesions was 5% for PGLs (adrenal PGLs: 7.9%) after pathological re-evaluation.

## Discussion

This study provides a comprehensive assessment of cystic and necrotic alterations in PGLs and their clinical significance. Cystic alterations were highly prevalent, affecting 61.8% of PGLs in our cohort. However, their presence did not correlate with differences in clinical presentation, regardless of the pathological presentation. In contrast, necrotic changes were uncommon (present in 3.9% of PGLs), but were associated with adverse clinical features. These included an increased rate of metastasis, a more symptomatic manifestations of the disease and more cardiovascular complications. Necrotic PGLs presented with a larger tumor volume and a predominantly noradrenergic phenotype, which likely underlies their more aggressive clinical behavior. Although information on necrotic alterations might be crucial for managing these patients, for example, in terms of preoperative preparation and follow-up, this information is not consistently reported in pathology and imaging reports. Our findings underscore the importance of systematically reporting necrosis, as its presence should be considered during follow-up, particularly given the increased metastatic tendency of necrotic PGLs. Adrenal and extra-adrenal PGLs, particularly HNPGLs, differ in their cellular origin, genetics, and glucocorticoid exposure, shaping their biochemical phenotype, presentation, and metastatic risk [178]. Therefore, we analyzed the full PGL cohort and adrenal PGLs separately to account for location-related differences (Table S5-6). While extra-adrenal tumor location has been previously described as an independent predictor of metastatic disease and disease-specific survival [179, 180], a particularly striking finding of this study was that all adrenal PGLs classified as necrotic after pathological re-evaluation were metastatic. This observation highlights necrosis as strong indicator of aggressive behavior, especially in adrenal PGLs. Overall, incidence of necrotic and cystic lesions was higher in adrenal than in extra-adrenal PGLs.

In our single center cohort, cystic lesions were reported in only 22.3% of PGLs cases in routine pathology/radiology reports. However, following systematic pathological re-evaluation the frequency of cystic alterations increased to 61.8%, revealing a substantial underestimation in standard clinical reporting. These findings are consistent with a previous study re-evaluating CT and/or MRI findings reporting cystic lesions in 55% of adrenal PGLs [16]. In contrast, another large cohort identified cystic adrenal PGLs in only 3.2% of cases [17]. Variability across different studies may reflect differences in sizes and morphologies of cysts/pseudocysts, inconsistent reporting and the resolution of detection method (imaging versus pathology). Furthermore, a distinction between pseudocysts and cysts can only be made through a rigorous pathological examination.

Previously, we demonstrated a higher prevalence of adrenal PGLs in the right adrenal [181], which was confirmed in our single-center study and the meta-analysis (56.5% vs. 53.5%). In line with our finding (66.7% cystic right-sided PGLs), a single center cohort identified predominantly right-sided PGLs with cystic changes (71%) [17] and no bilateral cases. Nevertheless, necrotic alterations were observed more frequently in left-side PGLs (55.6%). There were two bilateral cases, both exhibited necrosis. These findings suggest factors beyond anatomical prevalence, potentially including genetic determinants, might also play a role in initiating necrotic changes. However, genetic data were available for only two patients (*NF1* and *SDHC*), precluding definitive conclusions.

Although adrenal PGLs were more frequently diagnosed in females in European cohorts [181]; males more often exhibited germline PVs in cluster 1 genes and presented with higher rates of sympathetic PGLs and metastatic disease [182]. In our cohort, patients with necrotic PGLs were more often diagnosed in males and were associated with an increased metastatic rate. Notably, necrotic alterations were mostly observed in adrenal PGLs in our cohort (3 adrenal PGLs and 1 HNPGL). As adrenal PGLs carry a lower risk of metastasis than extra-adrenal PGLs [180], this finding identifies a clinically relevant subgroup of adrenal PGLs with unexpectedly aggressive behavior. Although histopathological scoring systems (e.g., PASS and GAPP) incorporate necrosis, but not cystic alterations, to stratify the risk of metastasis in PGLs, they remain limited in their ability to accurately predict metastatic disease [183]. The presence of necrosis in combination with biochemical, clinical and genetic markers previously discussed [179, 184], may therefore contribute to improve the prediction of metastatic disease in patients with PGLs.

Our meta-analysis revealed that necrotic PGLs were more often diagnosed due to catecholamine-related signs and symptoms compared to PGLs with cystic lesions. However, this was only in trend observed in our single center cohort comparing necrotic PGLs with PGLs without cystic/necrotic lesions. Dogra et al. demonstrated that biochemical abnormality in cystic adrenal PGLs correlate with solid, not cystic component [17]. In our cohort, plasma metanephrine levels were comparable between adrenal PGL patients with cystic and without cystic/necrotic lesions, while necrotic adrenal PGLs showed elevated plasma normetanephrine and methoxytyramine levels. Necrotic PGLs were associated with a higher number of symptoms and signs, confirming the finding that necrotic PGLs are more frequently diagnosed due to catecholamine-related signs and symptoms. They experienced an increased occurrence of weakness, sweating, and palpitations compared to patients with PGL without cystic/necrotic changes. The storage and secretion of catecholamines is strictly controlled in chromaffin cells [185]. Necrotic PGLs presented with a larger tumor volume and predominantly noradrenergic phenotype in our cohort, which is characterized by a less differentiated catecholamine secretion machinery [186] that might contribute to the clinical presentation of these tumors. Moreover, necrotic changes disrupt membrane function and may lead to an increased accumulation of catecholamines in the necrotic cavities so that the release to circulation might no longer be controlled. This could explain why patients with necrotic PGLs may experience severe fluctuations in symptoms. Ohara et al. describe a patient with adrenal PGL presenting with necrotic changes who experienced acute attacks of alternating hypertension and hypotension [131]. These attacks may be related to mechanical stress on the tumor, causing a spontaneous and uncontrolled release of catecholamines. This is also of relevance to the current discussion regarding preoperative therapy with α-adrenoreceptor antagonists in symptomatic patients with PGL [18]. Based on our findings, preoperative α-blockade should in particular be performed if necrotic changes are identified during imaging, as uncontrolled release of catecholamines from necrotic areas due to tumor manipulation during surgery can lead to severe cardiovascular crises. Nonetheless, prospective, multicenter studies are required to validate this and to establish definitive clinical guidelines.

Main limitations of this study are the retrospective nature of the study and inclusion of patients from a single center only. As a result, the number of patients in the individual comparison groups is limited, which restricts the statistical power of the study. This is particularly the case for PGLs with cystic and necrotic lesions, which limits the significance of the findings. To minimize evaluation bias two experts re-evaluated the pathological sections. However, corresponding pathological sections were not available for all patients. Moreover, necrosis may develop due to vascular compromise or rapid tumor growth, and over time can undergo liquefaction and resorption, particularly after hemorrhage, resulting in pseudocystic spaces without an epithelial lining. This temporal evolution may lead to overlapping morphological features and complicate the distinction between active necrosis and secondary pseudocystic change. In this study, no distinction was made between true cysts and pseudocysts. Further studies should distinguish between these conditions, as the temporal progression from necrosis to pseudocysts may have implications for the signs and symptoms associated with catecholamine excess. In the case of the meta-analysis, potential selection and inclusion bias was minimized by having two independent reviewers assess all publications and by strict adherence to PRISMA criteria (Supplementary Material 3). Including tumors described as ‘giant cystic PGLs’ likely introduced a bias in tumor volume, which may explain the discrepancy between our cohort and the meta-analysis regarding the relationship between tumor volume and necrosis. While our cohort showed an association between necrosis and larger tumor volume, the meta-analysis suggested the opposite. Case reports often overrepresent extreme cases, biasing results. Furthermore, many studies provided only limited information on the presence and characteristics of cystic or necrotic alterations, such as whether lesions represented true cysts or pseudocysts and about the extent of the lesion. In cases where both cystic and necrotic changes were reported, it often remained unclear which type of lesion predominated and whether true cysts or pseudocysts were detected, thereby limiting the interpretability of these findings.

In conclusion, this study provides novel insights into the prevalence and clinical significance of cystic and necrotic alterations in PGLs. While cystic changes are highly prevalent in PGLs they do not influence clinical presentation these patients. In contrast, necrotic lesions are comparatively rare, but are associated with more aggressive behavior, and increased risk for cardiovascular complications. Our findings emphasize the importance of recognizing necrotic changes as a potential marker of disease aggressiveness, especially in adrenal PGLs. Integrating this pathological feature more into clinical practice can enhance risk stratification and may guide preoperative management and follow-up strategies for patients with PGL.

## Supplementary Information

Below is the link to the electronic supplementary material.

Supplementary Material 1 - Meta-analysis (XLSX 62.3 KB)

Supplementary Material 2 (DOCX 82.1 KB)

Supplementary Material 3 (PDF 41.8 KB)

## Data Availability

All data are available upon request from the corresponding author.
