# Supplementary material for: Tumor Necrosis is Associated with an Increased Metastatic and Cardiovascular Risk in Paragangliomas: A Single-Center Series and Meta-Analysis Comparing Necrosis and Cystic Degeneration in Paragangliomas
Source: Endocr Pathol. 2026 Aug 22;37(1):34. doi: 10.1007/s12022-026-09931-1 (PMC13498522; doi:10.1007/s12022-026-09931-1)
Supplement: Supplementary file 2 [file 12022_2026_9931_MOESM2_ESM.docx]

**Supplemental material**

**Tumor necrosis is associated with an increased metastatic and cardiovascular risk in paragangliomas: A single-center series and meta-analysis comparing necrosis and cystic degeneration in paragangliomas**

Anna-Maria Wiejak^1^, Georgiana Constantinescu^1,2^, Gintare Zygiene^3^, Mercedes Robledo^4,5^, Christian G. Ziegler^1^, Christina Pamporaki^1^, Jens-Peter Kühn^6^, Ronald de Krijger^7,8^, Matthias Meinhardt^9^, Nicole Bechmann^3,10,*^

^1^ Department of Medicine III, University Hospital Carl Gustav Carus, Technical University Dresden, Dresden, Germany

^2^ Grigore T Popa University of Medicine and Pharmacy, Iasi, Romania^[1]^

^3^ Institute for Clinical Chemistry and Laboratory Medicine, University Hospital Carl Gustav Carus, Medical Faculty Carl Gustav Carus, Technische Universität Dresden, Dresden, Germany

^4^ Hereditary Endocrine Cancer Group, Human Cancer Genetics Program, Spanish National Cancer Research Centre (CNIO), Madrid, Spain

^5^ Centro de Investigación Biomédica en Red de Enfermedades Raras (CIBERER), Institute of Health Carlos III (ISCIII), Madrid, Spain

^6^ Institute and Policlinic for Diagnostic and Interventional Radiology, Faculty of Medicine and University Hospital Carl Gustav Carus, Technische Universität Dresden, Dresden, Germany

^7^ Princess Máxima Center for Pediatric Oncology, Utrecht, the Netherlands

^8^ Department of Pathology, University Medical Center Utrecht, Utrecht, the Netherlands

^9^ Department of Pathology, Faculty of Medicine Carl Gustav Carus, Technical University Dresden, Dresden, Germany

^10^ Experimental Endocrinology and tumor cell metabolism, Medical School OWL, Bielefeld University, Bielefeld, Germany

*Corresponding author: Nicole Bechmann, University Hospital Carl Gustav Carus, Medical Faculty Carl Gustav Carus, Technische Universität Dresden, Fetscherstrasse 74, 01307 Dresden, Germany; *Tel*: +49 351 458-19687; *E-mail address*: [Nicole.bechmann@uniklinikum-dresden.de](mailto:Nicole.bechmann@uniklinikum-dresden.de)

**1. Methods**

**1.1.** **Clinical data assessment**

Data included patient characteristics (sex and age); tumor information (location, date of first diagnosis, tumor dimensions, and occurrence of metastasis); mutation status (germline versus somatic PV and mutated gene); biochemical work up (plasma free normetanephrine, metanephrine and methoxytyramine), type of imaging and pathology reports. Furthermore, it was documented whether the diagnosis was based on catecholamine-related signs and symptoms or was incidental. Patients who were diagnosed during post-operative follow-up or follow-up of mutation carriers were grouped as being diagnosed incidentally. Before study inclusion, patients completed a standardized assessment documenting any signs or symptoms reported within the 30 days preceding study entry. These symptoms included headache, sweating, palpitations, tremor, pallor, flushing, panic/anxiety, nausea/vomiting, weakness, and constipation. Metastatic disease was diagnosed based on the presence of metastases in areas where chromaffin cells are typically not present, such as the lungs, bones, liver, and lymph nodes. In most cases, this was determined by functional imaging, such as ^123^I-MIBG or ^68^Ga-labeled DOTATATE.

**1.2. Genetic testing**

Initial testing for germline PVs in established PGL susceptibility genes was performed at the University Hospital Carl Gustav Carus Dresden. Tumors with an unknown genetic background were additionally tested for somatic PVs using customized panel sequencing (Illumina, San Diego, CA) at the Hereditary Endocrine Cancer Group at the CNIO in Madrid, Spain. PVs in *SDHA*, *SDHB*, *SDHC*, *SDHD*, *SDHAF2*, *VHL*, *FH*, and *EPAS1* were classified as cluster 1 PGLs, while PVs in *RET*, *NF1*, *HRAS*, and *FGFR1* were classified as cluster 2 PGLs [1]. Patients for whom no PV was found were defined as negative.

**1.3. Biochemical analysis**

Plasma samples of 41 patients with adrenal paragangliomas were available. Plasma free metanephrines and methoxytyramine were measured by liquid chromatography with tandem mass spectrometry (LC–MS/MS) as described elsewhere [2]. For some patients (n=3), only the catecholamine phenotype was available from the medical records (n_total_=44).

**1.4. Meta-analysis**

**1.4.1. Search strategies**

Source: PUBMED (https://pubmed.ncbi.nlm.nih.gov/)

Used browser: Mozilla Firefox

**1.4.2.	Search for pheochromocytomas and paragangliomas with cystic lesions and cystic+necrotic lesions**

**Search terms:** **("Cysts"[MeSH Terms:noexp] OR "cystic"[Title/Abstract] OR "cystical"[Title/Abstract] OR "cystically"[Title/Abstract] OR "cysts"[Title/Abstract] OR "cyst"[Title/Abstract]) AND ("Paraganglioma"[MeSH Terms] OR "paraganglioma"[Title/Abstract] OR "paragangliomas"[Title/Abstract] OR "phaeochromocytoma"[Title/Abstract] OR "pheochromocytoma"[Title/Abstract] OR "phaeochromocytomas"[Title/Abstract] OR "pheochromocytomas"[Title/Abstract])**

- Records identified: 908
- First Filter: Time range (years)= since 2000
- Records remaining: 587
  - Records excluded: 321
- Second filter: language= English and German only
- Records remaining: 527
  - Records excluded: 60
- Assessment for eligibility: Necrosis/cysts mentioned in publication
  - Records excluded for the following:
  - Records excluded (full text not retrievable): 20
  - Records excluded (animal subjects): 5
  - Records excluded (PGL): 24
  - Records excluded (metastasis only):0
  - Records excluded (no operation):5
  - Records excluded (no PGL cases):69
  - Records excluded (no distinguishable cases): 77
  - Records excluded (same cases described in two records): 1
  - Records excluded (minimal information not achieved): 33
  - Records excluded (compound PGL): 9
  - Records excluded (missing phenotype distinction): 5
  - Records excluded (no necrosis/cysts mentioned): 172
- **Eligible records**: 107

**1.4.3.	Search for pheochromocytomas and paragangliomas with necrotic lesions**

**Search terms: ("Paraganglioma"[MeSH Terms] OR "paraganglioma"[Title/Abstract] OR "paragangliomas"[Title/Abstract] OR "phaeochromocytoma"[Title/Abstract] OR "pheochromocytoma"[Title/Abstract] OR "phaeochromocytomas"[Title/Abstract] OR "pheochromocytomas"[Title/Abstract]) AND ("Necrosis"[Mesh:NoExp] OR "necrosis"[Title/Abstract] OR "necrotic"[Title/Abstract] OR "necrotising"[Title/Abstract] OR "necrotization"[Title/Abstract] OR "necrotize"[Title/Abstract] OR "necrotized"[Title/Abstract] OR "necrotizing"[Title/Abstract] OR "necrose"[Title/Abstract] OR "necrosed"[Title/Abstract] OR "necrosi"[Title/Abstract] OR "necrosing"[Title/Abstract] OR "necroses"[Title/Abstract])**

- Records identified: 509
- First Filter: Time range (years)= since 2000
- Records remaining: 362
  - Records excluded: 147
- Second filter: language= English and German only
- Records remaining: 336
  - Records excluded: 26
- Assessment for eligibility: Necrosis/cysts mentioned in report
  - Records excluded for the following: 294
  - Records excluded (Double records): 22
  - Records excluded (full text not retrievable): 19
  - Records excluded (animal subjects): 16
  - Records excluded (no PGL): 57
  - Records excluded (no operation): 4
  - Records excluded (no PGL cases): 70
  - Records excluded (no distinguishable cases): 34
  - Records excluded (pre-operative intervention): 4
  - Records excluded (minimal information not achieved): 28
  - Records excluded (compound PGL): 4
  - Records excluded (missing phenotype distinction): 1
  - Records excluded (no necrosis/cysts mentioned): 37
- **Eligible records: 40**

**1.4.4.**	**Exclusion/inclusion criteria and collected data**

Articles published before 2000 were excluded. For 39 publications, full text was not retrievable, which led to exclusion. Patients that were not operated were excluded due to uncertainty regarding the final diagnosis. Studies that aimed to assess the effects of specific interventions, such as embolization of PGLs or the use of specific inhibitors (e.g., sorafenib, which was reported to cause necrosis [3]), were excluded because the extent to which these interventions affect potential cyst or necrosis formation was uncertain and could introduce potential bias. Compound PGLs were also excluded. Studies that did not distinguish between other phenotypes, such as hemorrhage were excluded because differentiation was impossible. Cases that did not mention any necrotic or cystic change were excluded. Patients were excluded if other imaging forms besides MRI or CT scans indicated cystic or necrotic changes, such as ultrasound, MIBG, or PET scans (unless an additional CT or MRI scan was performed). We critically reviewed patients from the same center who were published in separate manuscripts for overlapping characteristics to exclude duplication. During further searches, additional cases were identified (n_necrotic_=7; n_cystic_=8) that could not be identified using PUBMED. These were added accordingly. A total of 134 patients with cystic PGLs (115 publications), 78 patients with necrotic PGLs (47 publications) and 23 patients with cystic and necrotic PGLs (24 publications) were included in the present meta-analysis (in total: 235 patients). Besides the requested minimal data, the following clinical features were collected: tumor side (left or right adrenal or bilateral), tumor volume, PV, cardiovascular complications, intraoperative complications, patient alive and mode of discovery (incidentally or based on catecholamine-related signs and symptoms).

**Table S1: Characteristics of patients with adrenal paragangliomas in dependence of occurrence of cysts or necrosis reported by routine radiological and/or pathological evaluation**

|  | **Total** | **Negative^a^** | **Cystic lesions** | **Necrotic lesions** | **Cystic and necrotic lesions** |
| --- | --- | --- | --- | --- | --- |
| **Number of patients n ([%])** | 59 (100) | 27 (45.7) | 21 (35.6) | 7 (11.9) | 4 (6.8) |
| **Number of tumors n ([%])^2^** | 63 (100) | 29 (46.0) | 21 (33.3) | 9 (14.3) | 4 (6.4) |
| **Sex^1^** Female n ([%]) Male n ([%]) | 30 (50.8) 29 (49.2) | 17 (62.9) 10 (37.0) | 11 (52.4) 10 (47.6) p<0.05^b^ | 1 (14.3) 6 (85.7) **p=0.0348**^b^ | 1 (25) 3 (75) |
| **Age at operation^1^** Mean ± SD [years] Median (range) [years] | 48.7 ± 16.5 50 (9-77) | 51.2 ± 15.4 53 (11-75) | 44.3 ± 17.4 45.5 (9-72) | 44.4 ± 17.7 51 (12-64) | 60.75 ± 12.3 59.5 (47-77) |
| **Tumor volume^2^** Mean ± SD [cm^3^] Median (range) [cm^3^] | 83.1 ± 145.5 41.4 (0.3 – 904.9) | 39.3 ± 50.0 21.8 (0.3-245.1) | 107.8 ± 194.8 46.8 (4.2 – 904.9) p<0.05^b^ | 151.1 ± 204.9 82.5 (18.9-659.8) **p=0.0108^b^** | 107.1 ± 77.6 99.0 (21.0-206.5) |
| **Tumor lateralization^2^** left:right n ([%]) bilateral cases n ([%]) | 27(43.5):35(56.5) 4 (6.9) | 13(46.4):15(53.6) 2 (7.7) | 7(33.3):14(66.7) 0(0) | 5(55.6):4(44.4) 2 (28.6) | 2(50):2(50) 0(0) |
| **Cysts/Necrosis described in:** imaging report n ([%]) pathology report n ([%]) both n ([%]) not mentioned n ([%]) | 5 (7.9) 15 (23.8) 14 (22.2) 29 (46.0) | 0 0 0 29 (100) | 2 (9.5) 7 (33.4) 12 (57.1) 0 | 2 (22.2) 6 (66.7) 1 (11.1) 0 | 1 (25) 2 (50) 1 (25) 0 |
| **Metastatic disease ^1^** yes:no n ([%]) | 4(6.8)/55(93.2) | 1(3.7)/26(96.3) | 0(0)/21(100) p<0.05^b^ | 3(42.9)^c^/4(57.1) **p=0.0211**^b^ | 0(0)/4(100) |
| **Mode of discovery n ([%)]** incidentally catecholamine-related SS post-OP follow-up genetic surveillance other reasons or no information | 28 (47.5) 29 (49.2) 0 0 2 (3.3) | 12 (44.4) 15 (55.6) 0 0 0 | 12 (57.1; p<0.05)^b^ 8 (38.1; p<0.05)^b 0^ 0 1 (4.8) | 4 (57.1; p<0.05)^b^ 3 (42.9; p<0.05)^b^ 0 0 0 | 0 3 (75) 0 0 1 (25) |
| **Pathogenic variants^1,3^**  n ([%]) | *EPAS1*: 2 (3.9) *FGFR1*: 1 (1.9) *FH*: 1 (1.9) *HRAS*: 4 (7.7) *NF1*:13 (25.0) *RET*: 5 (9.6) *SDHC*: 1^d^ (1.9) *VHL*: 5 (9.6) negative: 20 no information available: 7 Cluster 1: 9 (28.1)  Cluster 2: 23 (71.9) | *EPAS1*: 1 (4.3)  *FH*: 1 (4.3) *HRAS*: 2 (8.7) *NF1*: 8 (34.8) *RET*: 1 (4.3)  *VHL*: 2 (8.6) negative: 8 no information available: 4 Cluster 1: 4 (26.7)  Cluster 2: 11 (73.3) | *EPAS1*: 1 (5) *FGFR1*: 1 (5)  *HRAS*: 1 (5) *NF1*: 3 (15) *RET*: 4 (20)  *VHL*: 2 (10) negative: 8 no information available: 1 Cluster 1: 3 (25)  Cluster 2: 9 (75) | *NF1*:1 (20)  *SDHC*: 1^d^ (20)  negative: 3 no information available: 2 Cluster 1: 1(50)  Cluster 2: 1(50) | *HRAS*: 1 (25) *NF1*:1 (25)   *VHL*: 1 (25) negative: 1  no information available: - Cluster 1: 1 (33.3)  Cluster 2: 2 (66.7) |
| **Signs and symptoms^1^**  Hypertension n ([%]) Headaches n ([%]) Sweatiness n ([%]) Palpitations n ([%]) Tremor n ([%]) Pallor n ([%]) Flushing n ([%]) Panic n ([%]) Nausea n ([%]) Weakness n ([%]) Abdomen pain n ([%]) Chest pain n ([%]) Constipation n ([%]) Paroxysmal HP n ([%]) Diabetes n ([%]) | 38/53 (71.7) 24/50 (48.0) 27/50 (54.0) 21/50 (42.0) 6/50 (12.0) 12/50 (24.0) 12/50 (24.0) 13/50 (26.0) 10/50 (20.0) 18/50 (36.0) 6/50 (12.0) 4/50 (8.0) 4/50 (8.0) 22/51 (43.1) 12/53 (22.6) | 16/24 (47.8) 11/23 (47.8) 12/23 (52.2) 9/23 (39.1) 3/23 (13.0) 8/23 (34.8) 7/23 (30.4) 7/23 (30.4) 7/23 (30.4) 9/23 (39.1) 3/23 (13.0) 2/23 (8.6) 4/23 (17.4) 11/23 (47.8) 6/24 (25.0) | 13/19 (68.4) 9/18 (50) 10/18 (55.6) 8/18 (44.4) 3/18 (16.7) 3/18 (16.7) 3/18 (16.7) 5/18 (27.8) 2/18 (11.1) 4/18 (22.2) 2/18 (11.1) 2/18 (11.1) 0/18 (0) 6/18 (33.3) 3/19 (15.8) | **6/6 (100)** 3/6 (50) 4/6 (66.7) **4/6 (66.7)** 0/6 (0) 1/6 (16.7) 2/6 (33.3) 1/6 (16.7) 1/6 (16.7) **4/6 (66.7)** 1/6 (16.7) 0/6 (0) 0/6 (0) **3/6 (50)** **2/6 (33.3)** | 3/4 (75) 1/3 (33.3) 1/3 (33.3) 0/3 (0) 0/3 (0) 0/3 (0) 0/3 (0) 0/3 (0) 0/3 (0) 1/3 (33.3) 0/3 (0) 0/3 (0) 0/3 (0) 2/4 (50) 1/4 (25) |
| ^1^ Patients with multiple tumors where information regarding all tumors were available are only included once in the analysis ^2^ Including bilateral and multiple tumors ^3^ Patients tested for pathogenic variants (germline and/or somatic testing) ^a^ No cystic or necrotic alterations described by imaging and pathological report ^b^ ChiSquare test with Fisher´s Exact test compared to negative PGLs ^c^ Two of three metastatic cases were male ^d^ Including one case with *SDHC* promoter methylation HP: hypertension; SS: signs and symptoms | | | | | |

**Table S2: Characteristics of patients with extra-adrenal paragangliomas in dependence of occurrence of cysts or necrosis reported by routine radiological and/or pathological evaluation**

|  | **Total** | **Negative^a^** | **Cystic lesions** | **Necrotic lesions** | **Cystic and necrotic lesions** |
| --- | --- | --- | --- | --- | --- |
| **Number of patients n ([%])** | 44 (100) | 42 | 2 | 0 | 0 |
| **Number of tumors n ([%])^2^** | 57 (100) | 55 | 2 | 0 | 0 |
| **Sex^1^** Female n ([%]) Male n ([%]) | 31 (70.5) 13 (29.5) | 29 (69.0) 13 (30.9) | 2 (100) 0 (0) | NA | NA |
| **Age at operation^1^** Mean ± SD [years] Median (range) [years] | 47.3 ± 17.8 50 (12-79) | 46.3 ± 17.6 48,5 (12-79) | 68.5 ± 7.8 68.5 (63-74) | NA | NA |
| **Tumor volume^2^** Mean ± SD [cm^3^] Median (range) [cm^3^] | 15.6 ± 35.2 2.4 (0.004-205.9) | 15.8 ± 35.8 2.4 (0.004-205.9) | 8.6 ± 11.3 8.6 (0.66-16.6) | NA | NA |
| **Localisation** EA:HN | 11(25):33(75) | 32(76.2):10(28.8) | 1(50):1(50) | NA | NA |
| **Metastatic disease ^1^** yes:no n ([%]) | 4(9.1):40(90.9) | 4(9.5):38(90.5) | 0(0):2(100) | NA | NA |
| **Mode of discovery n ([%)]** incidentally catecholamine-related SS post-OP follow-up genetic surveillance other reasons or no information | 36 (81.8) 1 (2.3) 1 (2.3) 4 (9.1) 2 (4.5) | 35 (83) 1 (2.3) 1 (2.3) 4 (9.5) 1 (2.3) | 1 (50) 0 (0) 0 (0) 0 (0) 1 (50) | NA | NA |
| **Pathogenic variants^1,3^**  n ([%]) | *CDKN1B*: 1 (2.8) *HRAS*: 1 (2.8) *KIF1B*:1 (2.8) *SDHA*:2 (5.6) *SDHAF2*: 1 (2.8) *SDHB*: 6 (16.7) *SDHC*: 1 (2.8) *SDHC* (promoter methylation): 1 (2.8) *SDHD*: 6 (16.7) *VHL*: 1 (2.8) negative: 15 (41.7)^3^ | *CDKN1B*: 1 (2.9) *HRAS*: 1 (2.9)  *SDHA*:2 (5.8) *SDHAF2*: 1 (2.9) *SDHB*: 6 (17.6) *SDHC*: 1 (2.9) *SDHC* (promoter methylation): 1 (2.9) *SDHD*: 6 (17.6) *VHL*: 1 (2.9) negative: 14 (41.2)^3^ | *KIF1B*: 1 (50)         negative: 1 (50)^3^ | NA | NA |
| **Signs and symptoms^1^**  Hypertension n ([%]) Headaches n ([%]) Sweatiness n ([%]) Palpitations n ([%]) Tremor n ([%]) Pallor n ([%]) Flushing n ([%]) Panic n ([%]) Nausea n ([%]) Weakness n ([%]) Abdomen pain n ([%]) Chest pain n ([%]) Constipation n ([%]) Paroxysmal HP n ([%]) Diabetes n ([%]) | 14/16 (87.5) 10/16 (62.5) 6/14 (42.9) 1/14 (7.1) 1/14 (7.1) 1/14 (7.1) 2/14 (14.3) 2/14 (14.3) 1/14 (7.1) 2/14 (14.3) 2/14 (14.3) 3/15 (20) 1/14 (7.1) 1/14 (7.1) 4/18 (22.2) | 13/15 (86.7) 9/15 (60) 5/13 (38.5) 1/13 (7.7) 1/13 (7.7) 1/13 (7.7) 1/13 (7.7) 1/13 (7.7) 0/13 (0) 2/13 (15.4) 1/13 (7.7) 2/14 (15.4) 0/13 (0) 1/13 (7.7) 3/17 (17.6) | 1/2 (50) 1/2 (50) 1/2 (50) 0/2 (0) 0/2 (0) 0/2 (0) 1/2 (50) 1/2 (50) 1/2 (50) 0/2 (0) 1/2 (50) 1/2 (50) 1/2 (50) 0/2 (0) 1/2 (50) | NA | NA |
| ^1^ Patients with multiple tumors where information regarding all tumors were available are only included once in the analysis ^2^ Including bilateral and multiple tumors ^3^ Patients tested for pathogenic variants (germline and/or somatic testing) ^a^ No cystic or necrotic alterations described by imaging and pathological report HP: hypertension; SS: signs and symptoms; EA: extra-adrenal; HN: head and neck | | | | | |

**Table S3: Characteristics of patients with metastatic PGL**

| **Patient No.** | **Sex** | **Age at operation [years]** | **Pathogenic variant** | **Somatic/**  **germline** | **Cyst or necrosis** | **Re-evaluation** | **Location tumor** | **Location of metastasis** |
| --- | --- | --- | --- | --- | --- | --- | --- | --- |
| P1A | M | 34 | *SDHD* | Germline | Negative | Cystic | HN | Bone |
| P16 | F | 66 | *HRAS* | Somatic | Negative | Negative | Adrenal | Lymph note |
| P25 | M | 56 | Negative | Somatic | Necrosis | Necrotic | Adrenal | Lymph note |
| P54A | F | 47 | *SDHD* | Germline | Negative | Negative | HN | Multiple, including liver + bone |
| P64 | M | 17 | *SDHB* | Germline | Negative | Cystic | HN | Bone+liver |
| P95 | M | 33 | Negative | Somatic | Necrosis | Necrotic | Adrenal | Bone+liver |
| P107 | M | 31 | *SDHB* | Germline | Negative | Negative | Extra-adrenal | Skeletal system |
| P128A | F | 12 | No information | No information | Necrosis | Necrotic | Adrenal | Liver |
| HN: Head & neck | | | | | | | | |

**Table S4: Characteristics of patients with paragangliomas in dependence of occurrence of cysts or necrosis after pathological re-evaluation**

|  | **Total** | **Negative ^a^** | **Cystic lesions** | **Necrotic lesions** | **Cystic and necrotic lesions** |  |
| --- | --- | --- | --- | --- | --- | --- |
| **Number of patients n (%)** | 103 (100) | 30 (29.4) | 64 (61.8) | 4 (3.9) | 5 (4.9) |  |
| **Number of tumors n (%)** | 120 (100) | 40 (33.3) | 69 (57.5) | 6 (5) | 5 (4.2) |  |
| **Sex** Female [n] ([%]) Male [n] ([%]) | 61 (59.8) 42 (40.2) | 21 (70) 9 (30) | 38 (59.4) 26 (40.6) p>0.05^h^ | 1 (25) 3 (75) p>0.05^h^ | 1 (20) 4 (80) |  |
| **Age at operation** Mean ± SD [years] Median (range) [years] | 48.1 ± 17.0 50 (9-79) | 44.1 ± 15.8 48 (11-66) | 50.1 ± 17.3 53 (9-79) p>0.05^b^ | 35.5 ±18.3 37 (12-56) p>0.05^b^ | 56.6 ± 12.5 51 (47-77) |  |
| **Tumor volume** n Mean ± SD [cm^3^] Median (range) [cm^3^] | 118 51.1 ± 113.0 15.9 (0.004-904.9) | 38 19.6 ± 31.0 6.6 (0.004-133.5) | 69 53.7 ± 118.8 18.8 (0.065-904.9) p>0.05^b^ | 6 174.7 ± 256.4 53.6 (0.52-659.8) **p=0.0339^b^** | 5 106.2 ± 78.8 103.7 (20.9-209.5) |  |
| **Localization ^1^** A:EA:HN:A+EA | 62:16:41:1 | 12:7:20:1 | 40:9:20:0 | 5:0:1:0 | 5:0:0:0 |  |
| **Cysts/Necrosis described in:** imaging report^f^ pathology report^f^ imaging and pathology report^f^ pathological re-evaluation^g^ | 6 (5.0) 22 (18.3) 45 (37.5) 47 (39.2) | 1 (2.5) 8 (20.0) 29 72.5)  2 (5.0) | 3 (4.3) 9 (13.0) 14 (20.3) 43 (62.3) | 1 (16.7) 3 (50.0) 1 (16.7) 1 (16.7) | 1 (20.0) 2 (40.0) 1 (20.0) 1 (20.0) |  |
| **Metastatic disease ^1^** yes:no n (%) | 7(6.8):96(93.2) | 3(10):27(90) | 2(3.1):62(96.9) p>0.05^h^ | 3(75):1(25) **p=0.0124^h^** | 0(0):5(100) |  |
| **Mode of discovery n ([%)] incidentally catecholamine-related SS post-OP follow-up genetic surveillance other reasons or no information** | 64 (62.2) 30 (29.2) 1 (1) 4 (3.8) 4 (3.8) | 20 (66.7) 7 (23.3)  3 (10) | 40 (62.5) p>0.05^h^ 19 (29.7) p>0.05^h^ 1 (1.6) 1 (1.6) 3 (4.7) | 2 (50) p>0.05^h^ 2 (50) p>0.05^h^ | 2 (40) 2 (40)   1 (20) |  |
| **Pathogenic variants n (%)** | *CDKN1B*: 1 (1.1) *EPAS1*: 2 (2.3) *FGFR1*: 1 (1.1) *FH*: 1 (1.1) *HRAS*: 5 (5.6) *KIF1B*:1 (1.1) *NF1*: 13 (14.8) *RET*: 5 (5.7) *SDHA*: 2 (2.3) *SDHAF2*: 1 (1.1) *SDHB*: 6 (6.8) *SDHC*^d^: 3 (3.4) *SDHD*: 6 (6.8) *VHL*: 6 (6.8) negative: 35 no information available: 15 Cluster 1^e^: 27 (52.9) Cluster 2^e^: 24 (47.1) | *EPAS1*: 1 (4.3)  *FH*: 1 (4.3) *HRAS*: 1 (4.3) *KIF1B*:1 (4.3) *NF1*: 2 (8.7)  *SDHA*: 2 (8.7) *SDHAF2*: 1 (4.3) *SDHB*: 2 (8.7) *SDHC*: 1 (4.3) *SDHD*: 2 (8.7) *VHL*: 2 (8.7) negative: 7 no information available: 7 Cluster 1^e^: 12 (80) Cluster 2^e^: 3 (20) | *CDKN1B*: 1 (1.8) *EPAS1*: 1 (1.8) *FGFR1*: 1 (1.8)  *HRAS*: 4 (7.0)  *NF1*: 10 (17.5) *RET*: 4 (7.0)    *SDHB*: 3 (5.3) *SDHC*^d^: 1 (1.8) *SDHD*: 4 (7.0) *VHL*: 3 (5.3) negative: 25  no information available: 7 Cluster 1^e^: 12 (38.7) Cluster 2^e^: 19 (61.3) | *SDHB*: 1 (33.3)    negative: 2  no information available: 1 | *NF1*: 1 (20) *RET*: 1 (20)     *SDHC*: 1 (20)  *VHL*: 1 (20) negative: 1  no information available: - Cluster 1^e^: 2 (50) Cluster 2^e^: 2 (50) |  |
| **Signs and symptoms**  Hypertension n (%) Headaches n (%) Sweatiness n (%) Palpitations n (%) Tremor n (%) Pallor n (%) Flushing n (%) Panic n (%) Nausea n (%) Weakness n (%) Abdomen pain n (%) Chest pain n (%) Constipation n (%) Paroxysmal HP n (%) Diabetes n (%) | 52/69 (75.4) 34/66 (51.5) 33/64 (51.6) 22/64 (34.4) 7/64 (10.9) 13/64 (20.3) 14/64 (21.9) 15/64 (23.4) 11/64 (17.2) 20/64 (31.2) 8/64 (12.5) 7/65 (10.8) 5/64 (7.8) 23/65 (35.4) 16/71 (22.5) | 9/14 (64.3) 4/12 (33.3) 4/11 (36.4) 3/11 (27.3) 0/11 (0) 4/11 (36.4) 3/11 (27.3) 1/11 (9.1) 3/11 (27.3) 3/11 (27.3) 0/11 (0) 1/12 (8.3) 0/11 (0) 5/11 (45.5) 2/13 (15.4) | 36/47 (76.6) 27/46 (58.7) 25/45 (55.6) 16/45 (35.6) 6/45 (13.3) 8/45 (17.8) 8/45 (17.8) 12/45 (26.7) 8/45 (17.8) 14/45 (31.1) 7/45 (15.6) 5/45 (11.1) 5/45 (11.1) 15/46 (32.6) 12/50 (24.0) | 3/3 (100) 2/3 (66.7) 2/3 (66.7) 1/3 (33.3) 0/3 (0) 0/3 (0) 1/3 (66.7) 0/3 (0) 0/3 (0) **1/3 (33.3)** 0/3 (0) 0/3 (0) 0/3 (0) 1/3 (33-3) 0/3 (0) | 4/5 (80) 1/5 (20) 2/5 (40) 2/5 (40) 1/5 (20) 1/5 (20) 1/5 (20) 2/5 (40) 0/5 (0) 2/5 (40) 1/5 (20) 1/5 (20) 0/5 (0) 2/5 (40) 2/5 (40) |  |
| ^1^ Patients with multiple tumors where information regarding all tumors were available are only included once in the analysis ^2^ Including bilateral and multiple tumors ^3^ Patients tested for pathogenic variants (germline and/or somatic testing) ^a^ No cystic or necrotic alterations described by imaging and pathological report ^b^ Kruskal-Wallis rank sum test compared to negative ^c^ Both metastatic cases are male ^d^ Including one case with *SDHC* promoter methylation e Pathogenic variants in *CDKN1B* and *KIF1B* were not included in the cluster analysis  ^f^ If sections available confirmed by pathological re-evaluation ^g^ Re-evaluation of the pathological sections changed the findings, as cysts or necrosis were confirmed that had not been mentioned in the pathology or imaging reports ^h^ ChiSquare test with Fisher´s Exact test compared to negative A: adrenal; EA: extra-adrenal; HN: head & neck; HP: hypertension; SS: signs and symptoms | | | | | | |

**Table S5: Characteristics of patients with adrenal paragangliomas in dependence of occurrence of cysts or necrosis after pathological re-evaluation**

|  | **Total** | **Negative ^a^** | **Cystic lesions** | **Necrotic lesions** | **Cystic and necrotic lesions** |  |
| --- | --- | --- | --- | --- | --- | --- |
| **Number of patients n (%)** | 59 (100) | 11 (18.6) | 40 (67.8) | 3 (5.1) | 5 (8.5) |  |
| **Number of tumors n (%)** | 63 (100) | 13 (20.6) | 40 (63.5) | 5 (7.9) | 5 (7.9) |  |
| **Sex** Female n (%) Male n (%) | 30 (58.8) 29 (49.2) | 7 (63.6) 4 (36.4) | 21 (52.5) 19 (47.5) p>0.05^h^ | 1 (33.3) 2 (66.7) p>0.05^h^ | 1 (20.0) 4 (80.0) |  |
| **Age at operation** Mean ± SD [years] Median (range) [years] | 48.7 ± 16.5 50 (9-77) | 45.4 ± 16.2 45 (11-66) | 49.7 ± 16.3 51 (9-75) p>0.05^b^ | 33.7 ± 22.0 33 (12-56) p>0.05^b^ | 56.6 ± 12.5 51 (47-77) |  |
| **Tumor volume** n Mean ± SD [cm^3^] Median (range) [cm^3^] | 62 83:1 ± 145:5 41:4 (0.3-904.9) | 12 29.7 ± 25.2 27.4 (0.3-73.6) | 40 80.4 ± 147.6 40.8 (4.2-904.9) p>0.05^b^ | 5 209.6 ± 270.3 82.5 (18.8-659.8) p=0.0917^b^ | 5 106.2 ± 78.8 103.7 (20.9-209.5) |  |
| **Cysts/Necrosis described in:** imaging report^f^ pathology report^f^ imaging and pathology report^f^ pathological re-evaluation^g^ | 5 (8.5) 16 (27.1) 20 (33.9) 18 (30.5) | 1 ((9.1) 4 (36.3) 5 (45.5) 1 (9.1) | 3 (7.5) 8 (20.0) 13 (32.5) 16 (40.0) | 0 (0) 1 (33.3) 2 (66.7) 0 (0) | 1 (20.0) 2 (40.0) 1 (20.0) 1 (20.0) |  |
| **Metastatic disease ^1^** yes:no n([%]) | 4(6.8):55(93.2) | 1(9.1):10(90.9) | 0(0):40(100) p>0.05^h^ | 3(100):0(0) **p=0.0110^h^** | 0(0):5(100) |  |
| **Mode of discovery n ([%)]** incidentally catecholamine-related SS post-OP follow-up genetic surveillance other reasons or no information | 28 (47.4) 29 (49.2)  2 (3.4) | 4 (36.4) 7 (63.6) | 21 (52.5) p>0.05^h^ 18 (45) p>0.05^h^  1 (2.5) | 1 (33.3) p>0.05^h^ 2 (66.7) p>0.05^h^ | 2 (40) 2 (40)  1 (20) |  |
| **Pathogenic variants n (%)** | *EPAS1*: 2 (3.8) *FGFR1*: 1 (1.9) *FH*: 1 (1.9) *HRAS*: 4 (7.7) *NF1*: 13 (25.0) *RET*: 5 (9.6) *SDHC*^d^: 1 (1.9) *VHL*: 5 (9.6) negative: 20 no information available: 7 Cluster 1^e^: 9 (28.1) Cluster 2^e^: 23 (71.9) | *EPAS1*: 1 (3.8)  *FH*: 1 (1.9) *HRAS*: 1 (7.7) *NF1*: 2 (25.0)   *VHL*: 2 (9.6) negative: 2 no information available: 2 Cluster 1^e^: 4 (57.1) Cluster 2^e^: 3 (42.9) | *EPAS1*: 1 (4.8) *FGFR1*: 1 (4.8)  *HRAS*: 3 (14.3) *NF1*: 10 (47.6) *RET*: 4 (19.0)  *VHL*: 2 (9.5) negative: 15 no information available: 4 Cluster 1^e^: 3 (14.3) Cluster 2^e^: 18 (85.7) | negative: 2 no information available: 1 | *NF1*: 1 (20.0) *RET*: 1 (20.0) *SDHC*^d^: 1 (20.0) *VHL*: 1 (20.0) negative: 1 no information available: - Cluster 1^e^: 2 (50.0) Cluster 2^e^: 2 (50.0) |  |
| **Signs and symptoms**  Hypertension n (%) Headaches n (%) Sweatiness n (%) Palpitations n (%) Tremor n (%) Pallor n (%) Flushing n (%) Panic n (%) Nausea n (%) Weakness n (%) Abdomen pain n (%) Chest pain n (%) Constipation n (%) Paroxysmal HP n (%) Diabetes n (%) | 38/53 (71.7) 24/50 (48.0) 27/50 (54.0) 21/50 (42.0) 6/50 (12.0) 12/50 (24.0) 12/50 (24.0) 13/50 (26.0) 10/50 (20.0) 18/50 (36.0) 6/50 (12.0) 4/50 (8.0) 4/50 (8.0) 22/51 (43.1) 12/53 (22.6) | 6/10 (60) 3/9 (33.3) 4/9 (44.4) 3/9 (33.3) 0/9 (0) 4/9 (44.4) 3/9 (33.3) 1/9 (11.1) 3/9 (33.3.) 3/9 (33.3) 0/9 (0) 0/9 (0) 0/9 (0) 5/9 (55.6) 2/9 (22.2) | 26/36 (72.2) 19/34 (55.9) 20/34 (58.8) 15/34 (44.1) 5/34 (14.7) 7/34 (20.6) 7/34 (20.6) 10/34 (29.4) 7/34 (20.6) 12/34 (35.3) 5/34 (14.7) 3/34 (8.9) 4/34 (11.8) 14/35 (40.0) 8/37 (21.6) | 2/2 (100) 1/2 (50) 1/2 (50) 1/2 (50) 0/2 (0) 0/2 (0) 1/2 (50) 0/2 (0) 0/2 (0) 1/2 (50) 0/2 (0) 0/2 (0) 0/2 (0) 1/2 (50) 0/2 (0) | 4/5 (80) 1/5 (20) 2/5 (40) 2/5 (40) 1/5 (20) 1/5 (20) 1/5 (20) 2/5 (40) 0/5 (0) 2/5 (40) 1/5 (20) 1/5 (20) 0/5 (0) 2/5 (40) 2/5 (40) |  |
| ^1^ Patients with multiple tumors where information regarding all tumors were available are only included once in the analysis ^2^ Including bilateral and multiple tumors ^3^ Patients tested for pathogenic variants (germline and/or somatic testing) ^a^ No cystic or necrotic alterations described by imaging and pathological report ^b^ Kruskal-Wallis rank sum test compared to negative ^c^ Both metastatic cases are male ^d^ Including one case with SDHC promoter methylation e Pathogenic variants in *CDKN1B* and *KIF1B* were not included in the cluster analysis  ^f^ If sections available confirmed by pathological re-evaluation ^g^ Re-evaluation of the pathological sections changed the findings, as cysts or necrosis were confirmed that had not been mentioned in the pathology or imaging reports ^h^ ChiSquare test with Fisher´s Exact test compared to negative HP: hypertension; SS: signs and symptoms | | | | | | |

**Table S6: Characteristics of patients with extra-adrenal paragangliomas in dependence of occurrence of cysts or necrosis after pathological re-evaluation**

|  | **Total** | **Negative^a^** | **Cystic lesions** | **Necrotic lesions** | **Cystic and necrotic lesions** |
| --- | --- | --- | --- | --- | --- |
| **Number of patients n ([%])** | 57 (100) | 27 | 29 | 1 | 0 |
| **Number of tumors n ([%])^2^** | 44 (100) | 19 | 24 | 1 | 0 |
| **Sex^1^** Female n ([%]) Male n ([%]) | 31 (70.5) 13 (29.5) | 14 (73.7) 5 (26.3) | 17 (70.8) 7 (29.2) | 0 (0) 1 (100) | NA |
| **Age at operation^1^** Mean ± SD [years] Median (range) [years] | 45.7 ± 17.7 48 (12-79) | 40.3 ± 15.7 47 (12-66) | 51.2 ± 18.4 56 (17-79) | 41 ± 0 | NA |
| **Tumor volume^2^** Mean ± SD [cm^3^] Median (range) [cm^3^] | 15.6 ± 35.2 2.4 (0.004-205.9) | 14.9 ± 32.8 2.2 (0.004 – 133.5) | 16.7 ± 38.3 3.0 (0.07-205.9) | 0.52 ± 0 | NA |
| **Localisation** EA:HN | 11(25):33(75) | 4(21.1):15(78.9) | 7(29.2):17(70.8) | 0(0):1(100) | NA |
| **Metastatic disease ^1^** yes:no n ([%]) | 4(9.1):40(90.9) | 2(10.5):17(89.5) | 2(8.3):22(91.7) | 0(0):1(100) | NA |
| **Mode of discovery n ([%)]** incidentally catecholamine-related SS post-OP follow-up genetic surveillance other reasons or no information | 36 (81.8) 1 (2.3) 1 (2.3) 4 (9.1) 2 (4.5) | 16 (84.2) 0 (0) 0 (0) 3 (15.8) 0 (0) | 19 (79.2) 1 (4.2) 1 (4.2) 1 (4.2) 2 (8.3) | 1 (100) - - - - | NA |
| **Pathogenic variants^1,3^**  n ([%]) | *CDKN1B*: 1 (2.8) *HRAS*: 1 (2.8) *KIF1B*: 1(2.8) *SDHA*: 2 (5.6) *SDHAF2*: 1 (2.8) *SDHB*: 6 (16.7) *SDHC*: 1 (2.8) *SDHC* promoter methylation: 1 (2.8) *SDHD*: 6 (16.7) *VHL*: 1 (2.8) negative: 15 (41.7) | *KIF1B*:1 (7.1) *SDHA*: 2 (14.3) *SDHAF2*: 1 (7.1) *SDHB*:2 (14.3) *SDHC*: 1 (7.1)  *SDHD*:2 (14.3)  negative: 5 (35.7) | *CDKN1B*: 1 (4.8) *HRAS*: 1 (4.8)  *SDHB*: 3 (14.3)  *SDHC* promoter methylation: 1 (4.8) *SDHD*: 4 (19.1) *VHL*: 1 (4.8) negative: 10 (47.6) | *SDHB*: 1 (100) | NA |
| **Signs and symptoms^1^**  Hypertension n ([%]) Headaches n ([%]) Sweatiness n ([%]) Palpitations n ([%]) Tremor n ([%]) Pallor n ([%]) Flushing n ([%]) Panic n ([%]) Nausea n ([%]) Weakness n ([%]) Abdomen pain n ([%]) Chest pain n ([%]) Constipation n ([%]) Paroxysmal HP n ([%]) Diabetes n ([%]) | 18/22 (81.8) 12/22 (54.5) 8/20 (40) 1/20 (5) 1/20 (5) 1/20 (5) 3/20 (15) 2/20 (10) 1/20 (5) 3/20 (15) 2/20 (10) 3/21 (14.3) 1/20 (5) 1/18 (5.5) 4/24 (16.7) | 4/7 (57.1) 2/6 (33.3) 1/5 (20) 0/5 (0) 0/5 (0) 0/5 (0) 1/5 (20) 0/5 (0) 0/5 (0) 0/5 (0) 0/5 (0) 1/6 (16.7) 0/5 (0) 0/5 (0) 0/7 (0) | 13/14 (92.9) 9/15 (60) 6/14 (42.9) 1/14 (7.1) 1/14 (7.1) 1/14 (7.1) 1/14 (7.1) 2/14 (14.3) 1/14 (7.1) 3/14 (21.4) 2/14 (14.3) 2/14 (14.3) 1/14 (7.1) 1/13 (7.7) 4/16 (25) | 1/1 (100) 1/1 (100) 1/1 (100) 0/1 (0) 0/1 (0) 0/1 (0) 1/1 (1) 0/1 (0) 0/1 (0) 0/1 (0) 0/1 (0) 0/1 (0) 0/1 (0) 0/1 (0) 0/1 (0) | NA |
| ^1^ Patients with multiple tumors where information regarding all tumors were available are only included once in the analysis ^2^ Including bilateral and multiple tumors ^3^ Patients tested for pathogenic variants (germline and/or somatic testing) ^a^ No cystic or necrotic alterations described by imaging and pathological report HP: hypertension; SS: signs and symptoms; EA: extra-adrenal; HN: head and neck | | | | | |

**Table S7: Characteristics of patients with cystic or necrotic paragangliomas in dependence of tumor location (after pathological re-evaluation).**

|  | **Negative** | | **Cystic lesions** | | **Necrotic lesions** | |
| --- | --- | --- | --- | --- | --- | --- |
|  | **Adrenal** | **Extra-adrenal** | **Adrenal** | **Extra-adrenal** | **Adrenal** | **Extra-adrenal** |
| **Number of patients n (%)** | 11 (36.7) | 19 (63.3) | 40 | 24 | 3 | 1 |
| **Number of tumors n (%)** | 13 (32.5) | 27 (67.5) | 40 | 29 | 5 | 1 |
| **Sex**  Female n (%) Male n (%) | 7 (63.6) 4 (36.4) | 14 (73.7) 5 (26.3) | 21 (52.5)  19 (47.5) | 17 (71.0) 7 (29.0) | 1 (33.3) 2 (66.7) | 0 (0) 1 (100) |
| **Age at operation** Mean ± SD [years] Median (range) [years] | 45.4 ±16.2 45 (11-66) | 43.4 ± 15.9 48 (12-66) | 49.7 ± 16.3 51 (9-75) | 51.0 ± 19.4 56.5 (17-79) | 33.7 ± 22.0 33 (12-56) | 41 |
| **Tumor volume n** Mean ± SD [cm3] Median (range) [cm3] | 12 29.7 ± 25.2 27.4 (0.3-73.6) | 26 14.9 ± 32.8 (p=0.0170)^a^ 2.2 (0.004-133.5 | 40 80.4 ± 147.6 40.8 (4.2-904.9) | 29 16.7 ± 38.3 (p<0.0001)^a^ 3.1 (0.07-206) | 5 209 ± 270.3 82.5 (18.8-659.8) | 1 0.52 |
| **Metastatic disease ^1^** yes:no n([%]) | 1(9.1):10(90.1) | 2(10.5):17(89.5) | 0(0):40(100) | 2(8.3):22(91.7) | 3(100):0(0) | 0(0):1(100) |
| **Mode of discovery n ([%)]** incidentally catecholamine-related SS post-OP follow-up genetic surveillance other reasons or no information | 4 (36.4) 7 (63.6) 0 0 0 | 16 (100) p=0.0147^b^ 0 (0) p=0.0002^b^ 0 3 0 | 21 (53.8) 18 (46.2) 0 0 1 | 19 (95) p=0.0376^b^ 1 (5) p=0.0005^b^ 1 1 2 | 1 (33.3) 2 (66.7) 0 0 0 | 1 (100) 0 0 0 |
| ^a^ Kruskal-Wallis rank sum test compared to adrenal PGLs  ^b^ ChiSquare test with Fisher´s Exact test compared to adrenal PGLs SS: signs and symptoms | | | | | | |

**Table S8: Characteristics of patients with adrenal paragangliomas in dependence of the occurrence of cystic or/and necrotic lesions reported in literature – Meta-analysis**

|  | **Total** | **Cystic lesions** | **Necrotic lesions** | **Cystic and necrotic lesions** |
| --- | --- | --- | --- | --- |
| **Number of tumors n ([%])** | 178 (100) | 105 (59) | 52 (29.2) | 21 (11.8) |
| **Number of patients n ([%])** | 174 (100) | 103 (59.2) | 51 (29.3) | 20 (11.5) |
| **Sex^1^** Female n ([%]) Male n ([%]) | 108 (62.1) 66 (37.9) | 61 (59.2) 42 (40.8) | 35 (68.6) 16 (31.4) | 12 (60.0) 8 (40.0) |
| **Age^1^** Mean ± SD [years] Median (range) [years] | 46.9 ± 16.9 49 (3-86) | 50.0 ± 16.3 51 (4-86) | 41.9 ± 16.7 45 (10-80) **p<0.0036**^b^ | 42.9 ± 15.6 45.5 (3-64) p>0.05^b^ |
| **Tumor side** left:right:bilateral:unknown^1^ | 66(46.5):76(53.5):7:29 | 33(44.6):41(55.4):5:26 | 23(47):26(53):1:1 | 10(53):9(47):1:0 |
| **Tumor volume [n]** Total, mean ± SD [cm^3^] Total, median (range) [cm^3^] | 136 624.9 ± 1176.9 247.1 (4.8-10307.2) | 90 740.1 ± 1351.7 288.7 (4.8-10307.2) | 25 95.0 ± 113.6 58.7 (9.2-414.7) **p<0.0001**^b^ | 21 729.9 ± 894.2 377.0 (31.7-3002.1) p>0.05^b^ |
| **Metastatic disease ^1^** yes:no:unknown n ([%]) | 9(9):91(91):74 | 3(6):47(94):53 | 4(11.4):31(88:6):16 p>0.05^a^ | 2(13.3):13(86.7):5 p>0.05^a^ |
| **Mode of discovery** n([%]) incidentally Catecholamine-related SS insufficient information | 69 (58.5) 49 (41.5) 60 | 49 (66.2) 25 (3.8) 31 | 7 (43.8; p=0.001^a^) 19 (56.2; p=0.001^a^) 26 | 13 (72.2) 5 (27.8) 3 |
| **Pathogenic variants^1,2^** *SDHB VHL RET NF1* | 1 5 6 4 | 0 3 5 1 | 1 1 0 1 | 0 1 1 2 |
| **Cardiovascular complications^1^** yes:no:unknown n ([%]) | 31(37.3):52(62.7):90 | 9(19.6):37(80.4):57 | 18(78.3):5(21.7):28 **p<0.0001**^a^ | 6(40):9(60):5 p>0.05^a^ |
| **Intraoperative complications^1^** yes:no:unknown n ([%]) | 26(51):25(49):123 | 18(47.4):20(52.6):65 | 1(33.3):2(66.7):48 p>0.05^a^ | 7(77.8):2(22.2):11 p>0.05^a^ |
| **Patient alive^1,3^**  yes:no:unknown n ([%]) | 115(92.7):9(7.3):50 | 56(95):3(5):44 | 41(89.1):5(10.9):5 p>0.05^a^ | 18(94.7):1(5.3):1 p>0.05^a^ |
| ^1^ Bilateral case where information regarding both tumors were available are only included once in the analysis ^2^ For most of the cases no information about pathogenic variants were available ^3^ Within the follow-up period of the respective paper ^a^ ChiSquare test with Fisher´s Exact test compared to cystic lesions ^b^ Kruskal-Wallis rank sum test compared to cystic lesions SS: signs and symptoms | | | | |

**Table S9: Characteristics of patients with extra-adrenal paragangliomas in dependence of the occurrence of cystic or/and necrotic lesions reported in literature – Meta-analysis**

|  | **Total** | **Cystic lesions** | **Necrotic lesions** | **Cystic and necrotic lesions** |
| --- | --- | --- | --- | --- |
| **Number of tumors n ([%])** | 61 (100) | 30 (49.2) | 28 (45.9) | 3 (4.9) |
| **Number of patients n ([%])** | 61 (100) | 30 (49.2) | 28 (45.9) | 3 (4.9) |
| **Sex^1^** Female n ([%]) Male n ([%]) | 34 (55.7) 27 (44.3) | 19 (63.3) 11 (36.7) | 13 (46.4) 15 (53.6) | 2 (66.7) 1 (33.3) |
| **Age^1^** Mean ± SD [years] Median (range) [years] | 47.3 ± 15.2 47 (9-78) | 53.1 ± 13.3 52 (23-78) | 41.4 ± 14.8 43 (9-65) | 45.3 ± 20.3 55 (22-59 |
| **Tumor location** EA:HN^1^ | 52(85.2):9(14.8) | 28(93.3):2(6.7) | 21(75):7(25) | 3(100):0(0) |
| **Tumor volume [n]** Total, mean ± SD [cm^3^] Total, median (range) [cm^3^] | 41 529.6 ± 862.9 110.6 (10.8-4147.4) | 24 763.4 ± 1063.5 240.2 (10.8-4147.4) | 14 142.8 ± 157.5 68.2 (12.6-477.9) | 3 464.1 ± 123.0 410.3 (377.0-604.8) |
| **Metastatic disease ^1^** yes:no:unknown n ([%]) | 4(12.1):29(87.9):28 | 0(0):16(100):14 | 4(25):12(75):12 | 0:1:2 |
| **Mode of discovery** n([%]) incidentally Catecholamine-related SS insufficient information | 34 (80.9)) 8 (19.1) 19 | 22 (84.6) 4 (15.4) 4 | 9 (69.2) 4 (30.8) 15 | 3 (100) 0 0 |
| **Cardiovascular complications^1^** yes:no:unknown n ([%]) | 2(8):23(92):36 | 1(7.7):13(92.3):16 | 1(11.1):8(88.9):19 | 0(0):2(100):1 |
| **Intraoperative complications^1^** yes:no:unknown n ([%]) | 3(25):9(75):49 | 3(42.9):4(57.1):23 | 0(0):3(100):25 | 0(0):2(100):1 |
| **Patient alive^1,3^**  yes:no:unknown n ([%]) | 51(96.2):2(3.8):8 | 25(96.2):1(3.8):4 | 23(95.8):1(4.2):4 | 3(100):0(0):0 |
| ^1^ Bilateral case where information regarding both tumors were available are only included once in the analysis ^2^ For most of the cases no information about pathogenic variants were available ^3^ Within the follow-up period of the respective paper ^a^ ChiSquare test with Fisher´s Exact test compared to cystic lesions ^b^ Kruskal-Wallis rank sum test compared to cystic lesions SS: signs and symptoms | | | | |

**Table S10: Characteristics of patients with metastatic PGL in the meta-analysis**

| **Patient No.** | **Sex** | **Age [years]** | **Pathogenic variant** | **Cyst or necrosis** | **Location tumor** | **Location of metastasis** |
| --- | --- | --- | --- | --- | --- | --- |
| C-70 | F | 43 | not mentioned | Cyst | Adrenal | Liver |
| N-20 | M | 18 | not mentioned | Necrosis | Adrenal |  |
| N-46 | M | 37 | not mentioned | Necrosis | Extra-adrenal | Lymph nodes |
| N-59 | M | 40 | not mentioned | Necrosis | Extra-adrenal | Jugulodigastric and spinal lymph nodes |
| N-62 | M | 59 | not mentioned | Necrosis | Adrenal | Bone, liver |
| N-65 | M | 36 | EWSR1::CREM gene fusion | Necrosis | Extra-adrenal | Lung |
| N-67 | F | 25 | negative | Necrosis | Adrenal | Lymph nodes |
| N-71 | F | 34 | Not mentioned | Necrosis | Adrenal | No information |
| N-75 | F | 50 | Not mentioned | Necrosis | Extra-adrenal | Lung |
| C-108 | M | 37 | Not mentioned | Cyst | Adrenal | Liver plus multiple para-aortic, perirenal, and perisplenic enlarged lymph nodes and a small metastatic nodule in the right adrenal gland, and bone, lung |
| C-110 | M | 55 | Not mentioned | Cyst | Adrenal | Lesion size and microvascular invasion |
| B-16 | F | 17 | Not mentioned | Both | Adrenal | Liver |
| B-19 | F | 41 | Not mentioned | Both | Adrenal | Liver |

**Table S11: Characteristics of patients with cystic or necrotic paragangliomas in dependence of tumor location (excluding head and neck paragangliomas) reported in literature – Meta-analysis**

|  | **Cystic lesions** | | **Necrotic lesions** | |
| --- | --- | --- | --- | --- |
|  | **Adrenal** | **Extra-adrenal** | **Adrenal** | **Extra-adrenal** |
| **Number of tumors n ([%])** | 106 (79.1) | 28 (20.9) | 51 (70.8) | 21 (29.2) |
| **Number of patients n ([%])** | 104 (78.8) | 28 (31.2) | 50 (70.4) | 21 (29.6) |
| **Sex^1^** Female n ([%]) Male n ([%]) | 62 (59.6) 42 (40.4) | 18 (64.3) 10 (35.7) | 34 (68) 16 (32) | 11 (52.4) 10 (47.7) |
| **Age^1^** Mean ± SD [years] Median (range) [years] | 50.3 ± 16.5 51.5 (4-86) | 53.8 ± 13.4^a^ 53.8 (23-78) | 41.52 ± 16.7 44 (10-80) | 40 ± 16.2^a^ 40 (9-63) |
| **Tumor volume [n]** Total, mean ± SD [cm^3^] Total, median (range) [cm^3^] | 91 739.6 ± 1344.2 288.7 (4.8-10307) | 24 763.4 ± 1063.5^a^ 240.2 (10.8-4147.4) | 24 98.5 ± 114.6 60.8 (9.2-414.7) | 12 155.6 ± 167.5^a^ 74.6 (12.6-477.9) |
| **Metastatic disease ^1^** yes:no:unknown n ([%]) | 3(5.8):48(94.2):53 | 0(0):15(100):13 | 4(11.8):30(88.2):16 | 2(18.2):9(81.8):10 |
| **Mode of discovery** n([%]) incidentally Catecholamine-related SS insufficient information | 49 (66.2) 25 (33.8) 30 | 21 (84) p>0.05 ^b^ 4 (16) p>0.05^b^ 3 | 7 (26.9) 19 (73.1) 24 | 1 (50) p>0.05 ^b^ 1 (50) p>0.05^b^ 19 |
| **Cardiovascular complications^1^** yes:no:unknown n ([%]) | 9(19.1):38(80.9):57 | 1(7.7):12(92.3):15^b^ | 17(77.3):5(22.7):28 | 1(12.5):7(87.5):13^b^ |
| **Intraoperative complications^1^** yes:no:unknown n ([%]) | 18(46.2):21(53.8):65 | 3(42.9):4(57.1):21^b^ | 1(33.3):2(66.7):47 | 0(0):3(100):18^b^ |
| **Patient alive^1,2^**  yes:no:unknown n ([%]) | 57(95):3(5):44 | 24(96):1(4):3^b^ | 40(88.8):5(11.2):5 | 16(94.1):1(5.9):4^b^ |
| ^a^ Kruskal-Wallis rank sum test compared to adrenal PGLs; if not indicated otherwise no significant changes were observed  ^b^ ChiSquare test with Fisher´s Exact test compared to adrenal PGLs; if not indicated otherwise no significant changes were observed SS: signs and symptoms | | | | |

**Reference**

1.	Fishbein, L., et al., *Comprehensive Molecular Characterization of Pheochromocytoma and Paraganglioma.* Cancer Cell, 2017. **31**(2): p. 181-193.

2.	Eisenhofer, G., et al., *Reference intervals for plasma concentrations of adrenal steroids measured by LC-MS/MS: Impact of gender, age, oral contraceptives, body mass index and blood pressure status.* Clin Chim Acta, 2017. **470**: p. 115-124.

3.	Maksimovic, O., et al., *Evaluation of response in malignant tumors treated with the multitargeted tyrosine kinase inhibitor sorafenib: a multitechnique imaging assessment.* AJR Am J Roentgenol, 2010. **194**(1): p. 5-14.
